# Anthelmintic Agents from African Medicinal Plants: Review and Prospects

**DOI:** 10.1155/2022/8023866

**Published:** 2022-12-31

**Authors:** Jonathan Jato, Emmanuel Orman, Yaw Duah Boakye, Emelia Oppong Bekoe, Samuel Oppong Bekoe, Samuel Asare-Nkansah, Verena Spiegler, Andreas Hensel, Eva Liebau, Christian Agyare

**Affiliations:** ^1^Department of Pharmacognosy and Herbal Medicine, School of Pharmacy, University of Health and Allied Sciences, Ho, Ghana; ^2^Department of Pharmaceutics, Faculty of Pharmacy and Pharmaceutical Sciences, Kwame Nkrumah University of Science and Technology, Kumasi, Ghana; ^3^Institute for Pharmaceutical Biology and Phytochemistry, University of Münster, Münster, Germany; ^4^Department of Pharmaceutical Chemistry, School of Pharmacy, University of Health and Allied Sciences, Ho, Ghana; ^5^Department of Pharmacognosy and Herbal Medicine, School of Pharmacy, University of Ghana, Accra, Ghana; ^6^Department of Pharmaceutical Chemistry, Faculty of Pharmacy and Pharmaceutical Sciences, Kwame Nkrumah University of Science and Technology, Kumasi, Ghana; ^7^Institute of Integrative Cell Biology and Physiology, University of Münster, Münster, Germany

## Abstract

Soil-transmitted helminthiasis affects more than 1.5 billion people globally and largely remains a sanitary problem in Africa. These infections place a huge economic burden on poor countries and affect livestock production, causing substantial economic losses and poor animal health. The emergence of anthelmintic resistance, especially in livestock, and the potential for its widespread in humans create a need for the development of alternative therapies. Medicinal plants play a significant role in the management of parasitic diseases in humans and livestock, especially in Africa. This report reviews anthelmintic studies that have been conducted on medicinal plants growing in Africa and published within the past two decades. A search was made in various electronic databases, and only full articles in English were included in the review. Reports show that aqueous and hydroalcoholic extracts and polar fractions obtained from these crude extracts form the predominant (80%) form of the extracts studied. Medicinal plants, extracts, and compounds with different chemical groups have been studied for their anthelmintic potential. Polyphenols and terpenoids are the most reported groups. More than 64% of the studies employed in vitro assays against parasitic and nonparasitic nematode models. Egg hatch inhibition, larval migration inhibition, and paralysis are the common parameters assessed in vitro. About 72% of in vivo models involved small ruminants, 15% rodents, and 5% chicken. Egg and worm burden are the main factors assessed in vivo. There were no reports on interventions in humans cited within the period under consideration. Also, few reports have investigated the potential of combining plant extracts with common anthelmintic drugs. This review reveals the huge potential of African medicinal plants as sources of anthelmintic agents and the dire need for in-depth clinical studies of extracts, fractions, and compounds from African plants as anthelmintic agents in livestock, companion animals, and humans.

## 1. Introduction

Parasitic worms affect more than one-quarter of the world's population, with soil-transmitted helminthiases (STH) accounting for about 1.5 billion infections [1, 2]. STH is one of the neglected tropical diseases (NTDs) that affects mainly people living in regions of high poverty, without adequate sanitation, and in close contact with infectious vectors, domestic animals, and livestock [2, 3]. They occur globally in the tropics and subtropics, including the Americas, Asia, and sub-Saharan Africa. These areas are more impacted because of the low levels of development [2, 4].

Helminth infection is largely a sanitary problem and is associated with the human-animal food chain. Parasite eggs present in human faeces contaminate the soil where they embryonate and are taken back into the intestinal tract through poorly treated drinking water and foods [5]. This creates a vicious cycle of recurrent infections that is often difficult to break or interrupt [3, 6].

Although helminthiases have a low fatality rate, they have a huge impact on human health and livestock production. The severity of symptoms in humans depends on the worm burden and whether monospecific or mixed infections are involved [2, 7]. Whilst children constitute the most vulnerable group to worm infestation, pregnant women also suffer impaired immunity and a lower quality of life [5, 8–10].

Based on the location of the adult parasite in the body, helminthiases may clinically present as intestinal (whipworms, intestinal roundworms, and hookworms), or tissue (trematodes, hydatid tapeworms, and tissue roundworms) parasites [5, 11]. The most common and widespread intestinal nematodes in humans include *Ascaris lumbricoides*, *Ancylostoma duodenale*, *Necator americanus*, *Trichuris trichiura*, and *Strongyloides stercoralis*, which have been classified as soil-transmitted helminthiases [3, 12]. Mild symptoms include abdominal pain, nausea, diarrhoea, and loss of appetite, and in children, severe cases may lead to anaemia, eosinophilia, stunted growth, malnutrition, pneumonia, and poor physical and cognitive development [3, 13]. High-intensity infections could result in intestinal obstruction requiring surgery and death in cases of *Strongyloides stercoralis* [14].

Unlike for some viral and bacterial diseases, there are currently no vaccines developed for human intestinal parasites [5, 15, 16]. In livestock, however, the first vaccine (Barbervax®) against *H*. *contortus*, which is derived from an intestinal surface antigen of the nematode, has proven to be a sustainable control measure in small ruminants [17]. Control measures mainly include periodic deworming, health education, and improvements in environmental sanitation. Seasonal chemotherapy with synthetic anthelmintics remains the primary measure to eliminate or reduce infecting helminths. Health education helps to prevent reinfection, while improved sanitary conditions reduce egg transfer to soil [14].

Morbidity due to helminthiases has been greatly reduced by the annual or biannual mass drug administration (MDA) in vulnerable populations. The two benzimidazole drugs, mebendazole and albendazole, are the core agents recommended by the World Health Organization (WHO) for MDA in children of school age. Both drugs are effective, cheap, easy to administer, and have been used in large populations for several years with minor side effects [14]. Other classes of anthelmintic drugs available include macrocyclic lactones, imidazothiazoles, tetrahydropyrimidines, and amino-acetonitrile derivatives. Other drugs, including levamisole, pyrantel pamoate, niclosamide, ivermectin, and piperazine, have contributed immensely to tackling livestock and human parasites [18].

The increase in cost, availability, continual reinfection, emergence of drug-resistant parasites, adverse events associated with population-wide drug use [9, 17, 19], and lack of coverage for other infectious agents like *Strongyloides* have become major drawbacks to the success of anthelmintic chemotherapy [20]. These threats have spurred the quest to discover and develop new, innovative, sustainable, effective, safe, alternative, and complementary treatment options, mostly from natural products [21–24].

In Africa, about 80% of the population largely depend on traditional remedies for their primary healthcare needs [25–27]. Compared to orthodox medicines, these remedies are relatively accessible and cheaper, perceived to be safe and effective, and form part of folkloric practices [22, 24, 28]. Plants form the larger part of these traditional remedies and have historically been used in treating internal parasites and other diseases in humans and livestock [15, 29, 30]. They constitute a viable source of chemically diverse molecules with broad-spectrum activity and can be a ready means to combat parasite resistance. From January 1981 to September 2019, 71 new approved drugs were entirely derived from natural products, 14 as natural botanicals, and 356 as semisynthetic derivatives of natural molecules [31]. However, there is currently no anthelmintic drug product approved that has been developed from plant sources [18].

Even though the chemical constituents and mechanisms by which medicinal plants elicit the observed activities are less known [28, 32], technological advancement has reignited research using in vitro and in vivo assays to evaluate ethnopharmacological claims and, where possible, identify such chemical entities and their mechanisms [22, 28].

This review is unique in the sense that it gathers information on anthelmintic extracts, fractions, and compounds from African medicinal plants. It seeks to reveal the potential of African medicinal plants as sources of new anthelmintic molecules and alternative therapies against helminthiases.

Whereas the African continent has a huge natural resource pool that is widely used by local people, especially indigenous people, for the management of many disease conditions, the continent remains one of the hardest hits by intestinal parasites [2]. There is increasing research into natural products, especially medicinal plants, as sources of new antiparasitic agents. Despite efforts to gather the library of these plant products, be they extracts, fractions, or purified compounds [7, 23, 33], those available from African medicinal plants are scattered and limited to certain geographical regions. This review, therefore, sought to expand this pool of information and to create a clear picture of the situation as far as studies of anthelmintic agents from African medicinal plants are concerned. Here, we elaborate on the various studies that have been conducted on medicinal plants native to Africa and espoused on the very promising plant families and species.

### 1.1. Methodology

#### 1.1.1. Inclusion and Exclusion Criteria

For the scope of this review, full-text articles published in credible peer-reviewed journals, publishers, and repositories (see below) whose studies focused on the anthelmintic activities of medicinal plants that grow in Africa were included. Only articles written in English and published between January 2002 and December 2021 were included, no matter where the study was conducted.

Articles written before 2001 and after 2021 were excluded. Articles that focused on extracts, fractions, and/or compounds isolated from medicinal plants not growing in Africa were also excluded. Even though this review is extensive, it is not a systematic review. The review also significantly focused on gastrointestinal nematode-related studies than other types of helminthiases.

#### 1.1.2. Literature Search and Data Extraction

Articles were identified through literature searches in relevant electronic databases and search engines, including Scopus, Science Direct, Academic Journals, African Journals Online (AJOL), HINARI, BioMed Central, Google Scholar, JSTOR, and PubMed. Bibliographies of included articles were further searched, and pertinent, relevant information retrieved in primary searches was added. This search was conducted between April 2020 and April 2022.

Articles that were retrieved were independently screened by at least three authors, and those that met the inclusion criteria were selected for review.

Data were often obtained from relevant portions of the articles, including the “materials and methods” and “results” sections. The extraction focused on the botanical source of plant material, the nature of extracts, fractions, or compounds, and the type of assay employed, including in vitro and in vivo studies. The relevant measures of efficacy in the test system, including IC_50_, EC_50,_ LC_50_, and LD_50_, were used to assess the anthelmintic potential of the study samples. Mendeley Desktop (version 1.19.4, copyright 2008–2020, Mendeley Ltd.) was used to manage the citations.

The botanical identities of plants and their habitats in Africa were verified against information from https://www.worldfloraonline.org/search (formerly https://www.theplantlist.org) and https://plants.jstor.org/plants/browse.

## 2. Anthelmintic Resistance in Humans and Livestock

Parasite susceptibility to the existing anthelmintic drugs continues to rapidly decline, leading to the emergence of drug-resistant parasites. Several studies have reported the development and spread of resistance to all major classes of anthelmintics [34–36], especially in livestock and, to a lesser extent, in companion animals and humans [9, 37]. The main contributing factors to drug resistance include selective pressure induced by high treatment frequencies, single-drug regimens, preventive mass treatments, inadequate dosing, indiscriminate use, and overreliance on synthetic drugs to control helminthiases [18, 34, 38].

High-frequency preventive chemotherapy in humans and livestock, as a result of the high disease burden and limited number of anthelmintics, causes a reduction in worm refugia-enhancing mutations and resistance development [5, 21, 39]. Prolonged use of single drugs, for example, the use of ivermectin for Onchocerciasis control in West Africa and praziquantel against Schistosomiasis in Egypt, has been associated with widespread resistance [34].

The development and spread of drug-resistant traits at the molecular level have been well investigated in the model organism *C*. *elegans* [40] and the barber's pole worm (*H*. *contortus*) [41]. Mutations in genes coding for drug receptor sites or the expression of genes involved in drug efflux, detoxification, or amphidial drug uptake have for instance been reported as possible causes of drug resistance [42, 43]. Resistance to the benzimidazoles in trichostrongylid nematodes in ruminants has been ascribed to mutations in the isotype 1 *β*-tubulin gene (E198A, E198L, F167Y, and F200Y) [44–46].

Nematodes, generally upon hatching, undergo multiple larval developmental stages into adult worms [5], and this multistage cycle poses a challenge to drugs that target just a few stages. Broad-spectrum activity against egg hatching, larval metamorphosis, and adult worms is therefore an ideal requirement for anthelmintic agents [7].

The use of plant extracts may significantly delay and reduce the spread of resistance among parasite populations [47, 48]. These multicomponent systems with natural products from very different classes could interact with multiple developmental stages, help reduce natural selection pressures, and delay resistance development, which are typically found in such multitarget systems [49–51]. Selective treatment of individuals, multidrug therapy, and environmental parasite control strategies slow down the emergence of selective resistance alleles [38].

## 3. Anthelmintic Drug Development from Natural Products: Prospects and Challenges

Since the beginning of the use of modern anthelmintics era in the 1950s, only a handful of such drugs are available for use in humans [52]. The rate of anthelmintic drug development by the pharmaceutical industry has nosedived over the past four decades, partly due to high costs and low returns from investments in this area [16, 53–55]. Following the successful introduction of ivermectin in 1987 against onchocerciasis in humans [56], two other agents, namely emodepside and tribendimidine, are well advanced in human clinical trials against this disease [5, 57, 58]. On the other hand, monepantel, emodepside, and derquantel have recently been approved for use in livestock [16].

Natural products, including those from plants, animals, fungi, marine organisms, and bacteria, have been acclaimed as the panacea to synthetic drug discovery challenges [54, 59]. Many studies end with the evaluation of plant extracts, fractions, and some isolated compounds for anthelmintic activities [50], with no plant-derived compound currently in use as an anthelmintic drug in humans [18]. Pyrethrum, nicotine, and rotenone are some drug products of plant origin that have been used as antiparasitic agents in veterinary practice [60].

The discovery and development of anthelmintic agents from natural sources and the isolation and characterization of bioactive constituents have therefore become the end goal of research in this less-funded area [7, 60]. Whereas several efforts have been made towards the isolation and characterization of antiparasitic compounds from plant sources in the past two decades, little is seen in other organisms such as bacteria and fungi [7]. The disadvantage of the isolation approach, however, is the unavoidable loss of so-called pharmacological synergy or toxicological antagonism associated with multicomponent extracts and fractions [7].

Issues of availability of bioactive minor compounds from the plant material in sufficient amounts, stability, formulation, delivery, compatibility, and many years of development have also kept some promising plant molecules out of the market. Because of their bulky nature, semisynthetic measures to modify the chemical structures and properties of plant molecules have proven difficult and expensive [60].

Generally, medicinal plants have not competed favourably with orthodox medicines as anthelmintics. The expensive human clinical efficacy and safety trials and bureaucratic licensing procedures, accompanied by a limited drug market, disincentivize the pharmaceutical industry from plant-based anthelmintic product development. The yield and nature of phytoconstituents are also variedly influenced by environmental factors like climate, altitude, soil type, rainfall, and herbivore predation. This erratic and unpredictable outcome affects the establishment of consistent quality control measures and hence reduces the interest of pharmaceutical investors [7].

Proprietary issues of ownership, royalties, access, government charges, and patency for plant-based drugs further deflate the hopes of drug-producing companies, which have high expectations for investment returns [60]. The presence of “pan assay interference compounds,” often referred to as “PAINS,” is militating against the advancement of preliminary bioassays on plant extracts. PAINS is an unorganised group of promiscuous molecules that occur as unspecific hits in several enzyme assays and in vitro screenings. These subversive compounds have often been considered for optimisation steps but end up consuming a lot of resources of investigators [61]. The presence of these molecules in high-throughput screens, on the other hand, should be recognised as a cautious group requiring further assessment rather than an outright rejection as irrelevant [62].

Contrary to the popular notion, not all natural compounds are innocuous [7]. Often produced as defence mechanisms or in response to external stress, some natural products from plants are potentially toxic to humans and animals and can be deleterious to physiological functions [63, 64]. An investigation by Ali et al. [65], for instance, revealed that even though the crude saponins from aerial parts of *Achillea wilhelmsii* and *Teucrium stocksianum* have significant anthelmintic activities, they were cytotoxic in the brine shrimp assay. This, therefore, implies that any anthelmintic hit from plant extracts must be verified for its safety in mammalian cells [66] and, if possible, in living organisms.

With the emergence and rapid spread of multidrug resistance to existing synthetic anthelmintics, the prospects for drug development from natural sources remain high [67]. To attract investors, herbal anthelmintics must be developed to a stage where rigorous and reproducible quality control can be assured. Standardized extracts have a huge market potential due to the current drive for organic food supplements [60]. Multicomponent plant extracts may potentiate efficacy, counter toxicity, enhance bioavailability, or improve the stability of each other in formulations [68–71].

A more interesting addition to the herbal industry is the advancement in the genetic engineering of specific metabolic pathways [72]. Genetic modification and tissue cultures can increase the yield of target molecules and improve the turnover rate [59]. This also counters the risk of plant depletion through wild harvesting and reduces the impact of an unfavourable climate on raw materials [72].

## 4. Brief Comparison of In Vitro and In Vivo Anthelmintic Assays

Preliminary screening of natural products for pharmacological activities requires the use of validated methods to guarantee reproducible outcomes [73]. Since helminth infections are complex and often involve mixed parasites with varied lifecycles, models for testing for antiparasitic activities also widely differ between species. These investigations are grouped into the in vitro and in vivo techniques [74].

Most primary investigations of plant materials for anthelmintic activity employ in vitro bioassays [22, 74–76], which rapidly screen large numbers of samples, are simple in design, easy to perform, cheap to implement, require minimal ethical considerations, require a small quantity of samples, and quickly churn out reproducible results [49, 75, 77, 78]. These assays target various stages of the parasite lifecycle, including egg laying or hatching, larval development, migration, motility, motor paralysis, and lethality [5, 79]. The current gold standard for assessing the susceptibility of adult and larval worms to drugs is in vitro worm motility assays using read out by microscopy [53]. Some in vitro assays use nonparasitic worm models, whereas others involve the isolation of eggs or larvae from experimentally or naturally infected animal hosts and the growth of larvae in vitro, during which periods the test substances can be applied and activity evaluated [80].

The results of basic in vitro assays of test samples (“hits”) are confirmed by the higher test models, which are specialized in vitro and in vivo studies to define possible “lead” status [78]. It is however often difficult to reproducibly extrapolate results from in vitro investigations to in vivo activity owing to pharmacokinetics issues [81]. The growth and maintenance of parasitic nematodes for long periods outside the host is often a laborious, expensive, and slow process that hinders effective in vitro studies [82]. The versatility, availability, ease of culture maintenance, and high proliferation rate make *C*. *elegans*, a free-living, nonparasitic nematode, a suitable model for many nematocidal and mechanistic studies [40, 76, 82].

In vivo assays involve the use of whole animals and are models that remain close to the patient, which is the final target for drug development [73]. Studies involve the in vivo investigation of anthelmintic potential using animals infected with the relevant parasites. The outcomes of in vivo studies are influenced by the mode of administration, nature, and dose of the test substance, host organism, and parasite species involved. Faecal worm, egg count, worm shedding, and host immune response are usually the parameters evaluated. Egg counts evaluate the effect of treatment on adult parasite fecundity, whereas parasite load depicts the effect on larvae or adult worms. The density-dependent fecundity effect, however, limits the significance of the FECR assay [49, 53] and cannot be used for *Strongyloides spp*., whose eggs are not passed in stool but larvae.

Although in vivo studies provide superior and reliable outcomes for pharmacological screening due to the natural, biological, pharmacological, pharmacokinetic, and toxicological environments, they are expensive, slow for large-scale investigations, labour-intensive, and often bedevilled with ethical and animal welfare issues [73, 75].

## 5. General Overview of Anthelmintic Evaluation of African Medicinal Plants

From this review, it is evident that African medicinal plants have great potential as sources of anthelmintic agents. An ideal anthelmintic agent should have broad spectrum activity, affecting almost all stages of the lifecycle of the nematodes and sufficient safety. Whereas some studies that considered more than one parasite stage reported such broad spectrum activities, a few others reported disparities in efficacy against various forms of the parasites [83–85].

The majority (78%) of the studies or reports reviewed employed in vitro assays in evaluating the anthelmintic activities of medicinal plants. In vitro models mainly focused on the ability of drug candidates to inhibit egg hatching, larval migration, motility, larval development or exsheathment, and survival [86–94].

The in vitro test models involve parasitic nematodes such as *Haemonchus contortus*, *Ancylostoma caninum*, *Ascaris suum*, *Heligmosomoides bakeri*, *Heligmosomoides polygyrus*, *Trichostrongylus axei*, *Strongyloides papillosus*, *Trichuris ovis*, *Oesophagostomum columbianum*, and *Oesophagostomum venulosum* [95–97]. Nonparasitic earthworms, including *Pheretima posthuma*, *Lumbricus terrestris*, *Eisenia fetida*, and *Eudrilus eugeniae*, have also been used as in vitro models for studying the anthelmintic effects of many extracts [98–103], cited in [104]. The free-living nematode, *C*. *elegans*, continues to remain the most widely used non-parasitic test model for in vitro anthelmintic studies [105–110].

*H*. *contortus* and related gastrointestinal nematodes (GIN) of small ruminants are the most widely investigated organisms in in vivo models, whereas sheep and goats are the major animals in which such clinical investigations have been reported [86, 87, 111, 112]. There are few reports involving trials in pigs, chickens, goldfish, snails, rats, and mice [113–117]. The ability of test substances to reduce faecal egg count (FEC), a typical measure of effects on fecundity, and postmortem intestinal worm burden are the parameters measured in in vivo assays. A few other studies evaluated the physiological impact of test substances on haematological indices of host animals in addition to the antiparasitic investigations [95, 111, 116, 118–120]. There was no report cited that investigated the clinical efficacy of extracts or isolated compounds in human subjects, neither was any activity testing reported on commercially available herbal anthelmintic products from these medicinal plants. There is, therefore, is a need to clinically evaluate some of these plant products and establish quality parameters for their development into standardised remedies for helminthiases.

The anthelmintic activities reported vary widely depending on the plant species, type of extract, strain of nematode, and its parasitic stage of development. A similar observation was reported in a review of anthelmintic agents used in goats [74]. Most in vivo studies, however, produced lower efficacies compared to their in vitro counterparts regarding the same plant samples [74]. The effects of pharmacokinetic processes such as absorption and metabolism and the biological variations of host animals could be accountable for these observations [75]. Also, many of the studies report activity lower than that observed for the standard anthelmintic drugs often used as positive controls [121–124]. An in vitro study of *Carica papaya* extracts against the Indian earthworm *P*. *posthuma*, however, was reported to show better paralytic (*p* < 0.0001) and wormicidal (*p* < 0.0001) activity than albendazole [6].

Almost 80% of the studies evaluated aqueous or hydroalcoholic extracts evaluated aqueous or hydroalcoholic extracts of various plant materials including root barks, stem barks, flowers, seeds, and whole plants, oils, and latex or exudates (Table 1). A few organic extracts, fractions, and crude powdered plant materials (mostly as feed) have also been studied [65, 84, 112, 169, 172, 197]. This trend is expected since many studies seek to replicate traditional applications of the study materials.

The pharmacological potential of medicinal plants is attributed to their specific natural product composition, which can be influenced by various factors, including changes in environmental conditions [203]. To assess the pharmacological activities of individual constituents, they must first be isolated and characterized. The elucidated chemical structures provide grounds for quality control, structural modification, syntheses, elucidation of biosynthesis pathways, and quantitative structure-activity relations (QSAR) studies [7]. In this review, several bioactive anthelmintic compounds belonging to different biosynthetical classes have been isolated from medicinal plants native to Africa. Though there have been efforts to gather these antiparasitic phytochemical libraries [7, 23, 33], there exists no such profile for African medicinal plants. The majority of the studies reporting anthelmintic activities of medicinal plants focused on extracts or fractions with limited bioactive constituents [8]. Like the extracts, most of their activities have only been evaluated at the in vitro stage, with not much clinical reporting in animals or humans.

**Figure 1:**
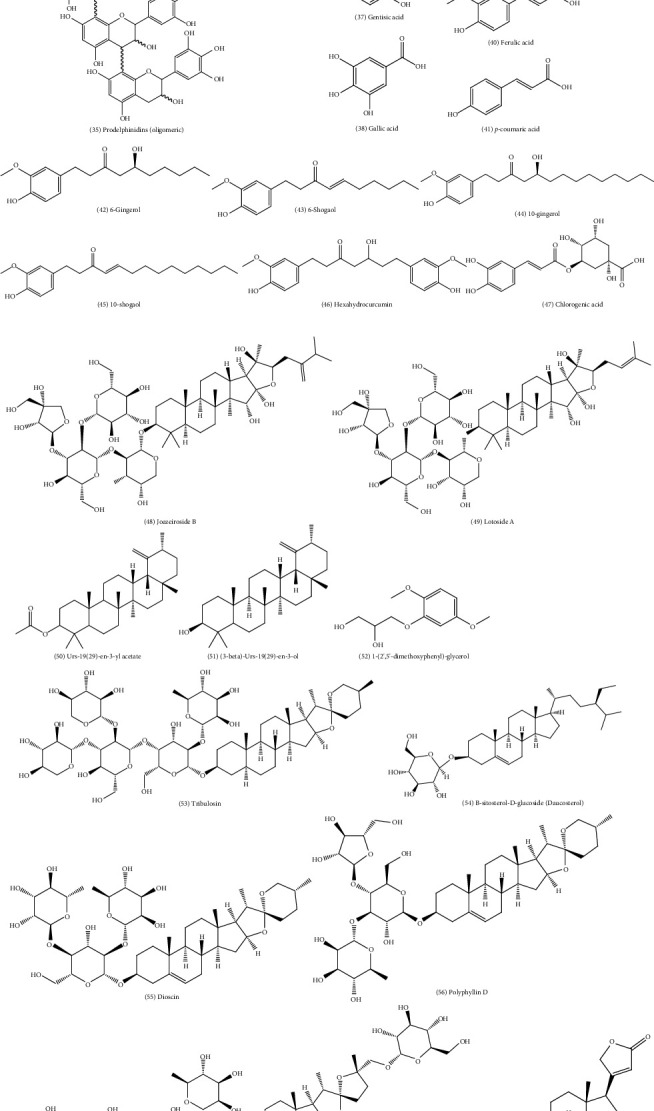
Chemical structures of compounds listed in Table 2 against helminthiases.

Some specific compounds isolated from plants have been reported to exhibit anthelminthic activity (Table 2 and Figure 1). Phenolic compounds such as tannins and flavonoids constitute a large class of natural molecules with potential anthelmintic or antiparasitic activities [74, 79, 244]. Because of their bulky structure and ability to bind several macromolecules, phenolic compounds have been reported to possess a broad range of biological activities. Oligomeric and polymeric proanthocyanidins, hydrolysable tannins, and flavonoids, for example, have been more intensively studied than any other class of natural anthelmintic compounds [79, 245]. A study conducted by Engström et al. [244] isolated and studied the in vitro activity of 33 hydrolysable tannins and gallic acid against egg hatching and larval motility of *H*. *contortus*. These compounds, isolated from various plants in Finland, showed varying anthelmintic activities [244]. Other studies isolated different types of proanthocyanidins from *P*. *pinnata* root bark and *C*. *mucronatum* leaves and reported varied activities against *C*. *elegans* and some animal intestinal parasites [150, 200]. Recent reviews on polyphenolics with anthelmintic potential have been published by Spiegler et al. [79] and Mukherjee et al. [246]. Alkaloids, coumarins, triterpenes, terpenoids, lignoids, prenylated derivatives, isothiocyanates formed after fermentation from glucosinolates, and saponins have also been widely isolated and studied [74]. Several fatty acids and aromatic compounds have also been reported to possess anthelmintic activities [247, 248]. Pineda-Alegría et al. [249] recently reported that long-chain fatty acids, including *β*-sitosterol, palmitic, pentadecanoic, stearic, and linoleic acids have nematocidal activity.

With the ever-increasing emergence of drug-resistant parasites and polyparasitism in animal and human helminthiases, there is a great need to explore the potential of developing some of the studied plants and their compounds into commercial drug products. The African herbal drug market should, therefore, explore the possibility of developing polyherbal formulations especially for use in livestock and companion animals, and as chemopreventive food supplements for humans.

## 6. Anthelmintic Activities of African Medicinal Plants

Medicinal plants belonging to different genera and families have been reported to have anthelmintic activities. However, some families have been frequently reported than others.

### 6.1. Fabaceae

This is the plant family with the highest reported number of plants with anthelmintic activities. *Acacia nilotica* (L.) Del. (*Fabaceae*), for instance, is a popular remedy for helminthiasis in Kenya [250]. Bachaya et al. [97] reported significant in vitro and in vivo activity of methanolic extracts of its fruits against the eggs (LC_50_ = 512.86 *μ*g/mL) and larvae (LC_50_ = 194.98 *μ*g/mL) of *H*. *contortus* and related ovine gastrointestinal nematodes. It also induced 78.5% reduction in FEC on day 13 post 3.0 g/kg treatment in sheep [97]. Similar effects were reported of its hydroalcoholic extracts against the larvae of *C*. *elegans* (wild type strain) and Onchocerca ochengi microfilariae with LC_50_ of 350 ± 1.1 *μ*g/mL and 10.8 ± 0.3 *μ*g/mL, respectively [110].

The aqueous extract of the root bark of *Albizia anthelmintica* Brongn, a plant traditionally used as an anthelmintic [251, 252], revealed potent in vitro ovicidal (ED_50_ of 144.2 *μ*g/mL) and larvicidal (ED_50_ of 65.2 *μ*g/mL) activities against strongyle nematodes of sheep [86]. Gathuma et al. [157] also reported 89.8% in vivo efficacy of similar extracts of the plant in FECR assays against mixed GIN infections in sheep.

Other *Acacia spp*. have been reported to possess promissory anthelmintic activities against various test models. This included the leaves of *A*. *polyacantha* Wild [112], *A*. *senegal*, *A*. *seyal*, and *A*. *tortilis* [141]. Extracts of other fabaceous plants, such as leaves of *Afrormosia laxiflora*, *Butea monosperma*, *Millettia ferruginea*, *Mimosa pudica*, *Senna occidentalis*, *Tephrosia spinosa*, *Tephrosia vogelii*, and *Tephrosia villosa*, stem barks of *Afzelia africana*, *Albizia schimperiana*, *Daniellia oliveri*, and the seed kernel of *Caesalpinia crista*, have all been reported to exhibit a varying spectrum of anthelmintic activities [88, 89, 94, 98, 109, 118, 127, 141, 145, 158].

### 6.2. Combretaceae

One important species in the family *Combretaceae* is *Anogeissus leiocarpus* (DC.) Guill. and Perr. (common name: Axlewood tree). It is widely used in African traditional practices and by livestock farmers for managing various parasitic disease conditions [89, 143, 145, 178]. Aqueous extracts of *A*. *leiocarpus* leaves caused 39.5% reduction in faecal egg count and 33% reduction in faecal worm burden in sheep treated with 400 mg/kg extract [143]. It also exhibited in vitro ovicidal (ED_50_ = 409.5 *μ*g/mL) and larvicidal (100% excludability inhibition at 1.2 mg/mL) actions against *H*. *contortus* [89], whereas its acetone extract inhibited egg hatch (LC_50_ = 360 *μ*g/ml) and larval development (LC_50_ = 509 *μ*g/ml) [92]. Ndjonka et al. [144] reported that an ethanolic extract of *A*. *leiocarpus* bark was more active than an aqueous extract with LC_50_ of 380 *μ*g/ml, significantly retarding the development of larvae into adult worms [144]. Another study reported significant in vitro activity of methanol and DCM extracts of leaves, roots, and bark against larvae of Rhabditis pseudoelongata with EC_50_ between 2.5 and 10 *μ*g/ml [145].

*Combretum mucronatum* Schumach and Thonn, traditionally used for various ailments including helminthiases in Africa [149, 253], has also been reported to exhibit anthelmintic activities against various test models. Ethanolic extract of *C*. *mucronatum* leaves exhibited in vitro nematocidal effects with 10 *μ*g/mL minimum lethal concentration against *T*. *muris* and induced 85.3% reduction of worm burden in mice [148]. Hydroalcoholic extracts also inhibited *C*. *elegans* larvae with LC_50_ of 1.67 mg/mL. A partition of this extract revealed that the ethyl acetate portion possessed stronger anthelmintic activity than the remaining aqueous fraction. The respective activity can be related to the presence of oligomeric proanthocyanidins with different structures [150]. Subsequent ultrastructural studies showed that the tannin-rich extract caused visible effects on the cuticle without overt effects on the intestines/gut of the worms [151].

Other plants from this family with reported potential anthelmintic activities include *Anogeissus schimperi*, *Combretum mole*, *Guiera senegalensis*, *Terminalia catappa*, and *Terminalia* glaucescens [106, 145–147, 153].

### 6.3. Cucurbitaceae

Some plants belonging to this taxonomic family have also been investigated for anthelmintic effects. Ethanolic extracts of *Momordica charantia* leaves collected from different ecological zones in Togo exhibited varying degrees of inhibition against *C*. *elegans* larvae with LC_50_ values between 473 and 997 *μ*g/ml [155]. Similar extracts of its fruits also caused 100% mortality of GIN larvae at 100 mg/mL in vitro and 78% FECR on day 9 posttreatment of goats with 100 mg/kg [126]. Aqueous and ethanolic extracts of seeds of *Citrullus lanatus*, *Cucurbita pepo*, and *Telfairia occidentalis* all exhibited significant mortality and paralysis in vitro against the earthworm *Lumbricus terrestris* at 50 mg/mL [154].

### 6.4. Lamiaceae

Some plants belonging to the *Lamiaceae* have been reported to possess anthelmintic properties. These include *Ocimum sanctum* L., whose essential oils and eugenol inhibited *C*. *elegans* larvae with ED_50_ of 62.1 *μ*g/mL [108]. The aqueous extracts of roots of *Leonotis ocymifolia* (Burm.f.) Iwarsson and aerial parts of *Leucas martinicensis* (Jacq) R.Br. showed ovicidal (ED_50_ = 0.25 *μ*g/mL and ED_50_ = 0.09 *μ*g/mL, respectively), and larvicidal (100% and 99.85% inhibition, respectively, at 50 mg/mL) effects against *H*. *contortus* [158]. Whereas the fruits of *O*. *basilicum* L. were active against the earthworm *E*. *eugeniae* [159], the leaves of *O*. *gratissimum* and essential oils of *Thymus bovei* Benth., respectively, inhibited *H*. *placei* (LC_50_ of 17.70 mg/mL) [160] and *P*. *posthuma* (*μ*g/mL) [161].

### 6.5. Meliaceae

*Azadirachta indica* A. Juss. (neem) is a widely known plant in African traditional medicine and contributes immensely to the management of livestock diseases and pests [254–256]. Almost every part of this plant has been reported to have anthelmintic activity. Polar extracts of neem seeds exhibited significant ovicidal and larvicidal action in vitro against *H*. *contortus*, with the ethyl acetate fraction causing 83% wormicidal effects at 50 mg/ml 1 h postexposure [162]. Aqueous and methanolic extracts of the seeds induced 29.3% and 40.2% reduction in EPG in sheep naturally infected with *H*. *contortus* and *Trichostrongyus spp*. on day 15 posttreatment with 3 g/kg [163]. Neem leaves also exhibited in vitro anthelmintic effects against the earthworm *Pheretima posthuma*, the tapeworm *Raillietina spiralis*, and the roundworm *Ascaridia galli* [164]. In vivo studies of feed in sheep reported significant inhibition of bovine nematodes, causing 98% reduction in FEC on day 14 posttreatment [165]. The leaf extracts also inhibited microfilariae of Setaria cervi in vitro [166] and caused a significant reduction in FEC and TWC against *H*. *polygyrus* in mice [84]. Sujon et al. [126] reported a 100% in vitro mortality at 100 mg/mL against GIN with 81% reduction in EPG on day 9 posttreatment of goats [126]. Leaves, stems, and root barks extracts inhibit strongyle nematodes, causing over 90% mortality of larvae at 100 mg/mL [167].

Another medicinally relevant species from the family Meliaceae is *Khaya senegalensis* (Mahogany). Ethanolic extract of *K*. *senegalensis* bark induced in vitro LC_50_ of 0.51 mg/mL and 88.82% FECR at 500 mg/kg in sheep against strongyle nematodes [168]. Similar extracts of the bark and leaves had LC_50_ of 470 *μ*g/mL and 1.0 mg/mL, respectively, against *C*. *elegans* larvae [144]. Methanol-dichloromethane extract of the whole plant of *Lansium domesticum* also inhibited adult *C*. *elegans*, significantly reducing their survival to 59% [107].

### 6.6. Musaceae

*Musa spp*. is the only genus in this family that has been reported to possess anthelmintic properties. Species such as *M*. x *paradisiaca*, *M*. *sapientum*, and *M*. *nana* inhibited the sheep tapeworm (*Moniezia benedeni*), roundworm (*Ascaris lumbricoides*), and adult earthworm (*Esenia fetida*), with *M*. x *paradisiaca* exhibiting the highest activity against the three worms [173]. *M*. x *paradisiaca* also caused significant FECR when fed to lambs infected with *H*. *contortus* [172] and in vitro ovicidal effects (LC_50_ = 2.13 *μ*g/mL) against the same parasite [181]. Other preparations of various parts of *Musa spp*. demonstrated ovicidal activities against *T*. *colubriformis* in sheep [169], in vitro ovicidal and larvicidal effects against *H*. *contortus*, and reduction of FEC in sheep infected with *H*. *contortus* [170, 171].

### 6.7. Rubiaceae

*Morinda lucida* and *Nauclea latifolia* are two plants from this family for which anthelmintic potential has been widely reported. Methanol and DCM extracts of leaves and roots of *M*. *lucida* inhibited larvae of *R*. *pseudoelongata* with EC_50_ of 2.5 *μ*g/mL [145], whereas hydroethanolic extracts of the stem bark induced dose-dependent paralysis (18.17 ± 0.03 min) and death (24.34 ± 0.21 min) at 50 mg/mL against *P*. *posthum*a [176]. Ethanolic extract of *M*. *lucida* leaves demonstrated concentration-dependent ovicidal action against *T*. *colubriformis* [177]. The aqueous and ethanolic extracts of *N*. *latifolia* leaves induced ovicidal activities (LC_50_ of 0.704 and 0.650 mg/ml, respectively) against ovine GIN and reduced faecal egg count when administered to naturally parasitised sheep [95]. Onyeyili et al. [178] reported significant reduction (93.8%) in FEC when sheep, infected with nematodes were treated with 1600 mg/kg body weight of aqueous extract of *N*. *latifolia* stem bark for 5 consecutive days [178]. Ethanolic extract of *Canthium mannii* stem bark induced 90% inhibition of egg hatching against *Ancylostoma caninum* at 1 mg/mL after 48 h incubation [96].

### 6.8. Other Plant Species with Anthelmintic Activities

#### 6.8.1. *Carica papaya* L. (*Caricaceae*)

Although the only plant in this family whose anthelmintic activities have been reported, *Carica papaya* (pawpaw) is one African medicinal plant whose anthelmintic potential has been widely investigated. Investigations on various extracts and parts of this plant have all reported some level of anthelmintic activity. Latex exudate from unripe fruits of *C*. *papaya* significantly reduced FEC (77.7%) of *Ascaridia galli* and *Cappilaria spp*. in poultry after one week of treatment [186]. After one week of posttreatment with 100 mg/mL, aqueous extracts of papaya seeds caused 100% reduction in FEC of GIN in goats [187] and sheep [188]. The seed extract also induced 100% reduction in FEC two weeks posttreatment in chicks [116] and goats [111]. In vitro studies reported that *C*. *papaya* seed extracts caused significant paralysis and death of adult *P*. *posthuma* [142] and inhibited egg hatch, larvae, and adult worms of *T*. *colibriformis* [177]. An in vitro comparative study of the leaves, stem bark, and seeds extracts of *C*. *papaya* reported that the seed extracts were the most active against adult *P*. *posthuma* [6]. Aqueous extract of papaya seeds was again reported to have more active LD_50_ of 49.94 and 49.32 mg/ml against *H*. *contortus* egg hatch and larval development, respectively [94]. The anthelmintic activity of pawpaw can be related to the isothiocyanates, which are formed from the genuine glucosinolates.

#### 6.8.2. *Vernonia amygdalina* Del. (*Asteraceae*)

*Vernonia amygdalina* (bitter leaf) is an important vegetable in West and Central African dishes [99] and widely used in the treatment of intestinal worms across Africa [253, 254]. Anthelmintic studies of aqueous and ethanolic extracts of its leaves revealed significant paralytic effects (59.94 ± 8.25 and 33.18 ± 12.41 min, respectively) against the adult earthworm *L*. *terrestris* [99]. Acetone extract of *V*. *amygdalina* leaves exhibited 42% ovicidal effect, 70% larval migration inhibition and 90% adulticidal effects at 300 *μ*g/mL against *H*. *contortus* [141]. Alawa et al. [131] reported no significant ovicidal effects of *V*. *amygdalina* leaves extract at 11.2 mg/mL against *H*. *contortus* [131]. Another study revealed that the chloroformic extract of the stem bark was more active against *P*. *posthuma*, inducing paralysis (11.95 ± 0.28 min) and death (41.74 ± 2.21 min), than its ethanolic counterpart [142]. The anthelmintic activity might be related to the presence of sesquiterpene lactones.

#### 6.8.3. *Garcinia kola* Heckel (*Clusiaceae*)

Commonly referred to as “bitter kola,” *Garcinia kola* is used to treat gastrointestinal helminthiases [190] and has been shown to possess this activity in pharmacological screenings. Hydroethanolic extract of *G*. *kola* seeds induced 76.5% irreversible paralysis of *H*. *bakeri* larvae at 50 mg/mL [91] whilst its aqueous extract exhibited 53.3% larvicidal effects against strongylid nematodes of goats at same concentration [189]. At 50 mg/mL, the stem bark extract induced dose-dependent paralysis and death of the adult *P*. *posthuma* at 39.29 ± 0.12 and 54.29 ± 0.01 min, respectively, for 50 mg/mL [176]. Both the seed and stem bark extracts were ovicidal (98.9% and 100%, respectively) at 100 mg/mL against strongylid nematodes [190].

#### 6.8.4. *Paullinia pinnata* L. (*Sapindaceae*)

*Paullinia pinnata* is used in sub-Saharan Africa as an anthelmintic agent, especially for treating ancylostomiases [149, 258]. The anthelmintic properties of its root bark and leaves have been explored, revealing a huge potential as a source of nematocidal molecules, mainly oligomeric proanthocyanidins in the bark. Okpekon et al. [145] reported that extracts of both leaves and root bark of *P*. *pinnata* have in vitro inhibitory effects on *R*. *pseudoelongata* with EC_50_ of 2.5 *μ*g/ml each [145]. The hydroethanolic extract of the root bark also reduced the survival of *C*. *elegans* larvae to 85.2% at 1 mg/mL [149]. Further in vitro investigations of this extract against some animal parasites and *C*. *elegans* revealed that the extract had significant activity against *C*. *elegans* (LC_50_ = 2.5 mg/mL), *Toxocara cati* (LC_50_ = 112 *μ*g/mL) and *Trichuris vulpis* (LC_50_ = 17 *μ*g/mL) [199]. Fractionation of water-acetone extracts leads to an ethyl acetate partition with better anthelmintic activity (LC_50_ = 1.1 mg/mL) than the water fraction (LC_50_ = 2.9 mg/mL) and the crude extract (LC_50_ = 1.9 mg/mL) [200]. Bioassay-guided studies led to the isolation of Cinnamtannin B1, a trimeric A-type procyanidin, which had significant inhibition of *C*. *elegans* (86.5% at 72 h incubation). The respective *B*-type trimer, procyanidin *C*1, isolated from *C*. *mucronatum*, was less active (47.3%), indicating a strong influence of the interflavan linkage and the different fine structures of the procyanidins [150, 200].

## 7. Conclusion

The World Health Organization's 2030 targets for STH can only be achieved with renewed investments in new and effective drugs. African medicinal plants will serve as a useful source of remedies for integrated parasite control, along with other measures such as education and the provision of sanitation facilities to at-risk populations.

The foregoing data validate the claims that African medicinal plants have huge potential for the discovery and development of new, innovative, and alternative anthelmintic agents. The majority of reports and studies evaluated extracts of plants, with a few isolated compounds also characterized. Even though in vivo animal studies abound, 78% of the studies reported in vitro activities against parasitic nematodes. There are no reports available on clinical investigations of extracts or purified compounds in humans, nor have any commercial products been reported or evaluated for their effectiveness. Therefore, clinical evaluation of these plant products and mechanistic studies, especially on isolated compounds, will advance the goal of identifying and developing drug candidates from plant sources. [256].

## Figures and Tables

**Table 1:** African medicinal plants with anthelmintic activities, sorted according to the respective plant families.

No.	Nature of extract	Botanical source	Habitat in Africa	Assay(s) conducted	Outcome of assay(s)
*Acanthaceae*					
(1)	Aqueous extract	Leaves of *Acanthus montanus* (Nees) T. Anders	West and East Africa	In vitro EHIA, and larval growth inhibition assay against strongylid nematodes of sheep and goats	91.75% reduction in egg hatch and 67.02% larval inhibition at 25 mg/ml in 24 h [90]
(2)	1 : 1 DCM: methanolic extract	Roots and leaves of *Linariantha bicolor* B. L. Burtt & R. M. Sm.	Tropical Africa	In vitro activity against *L*3, *L*4 and adult *C*. *elegans* (wild type, Bristol *N*2)	Root extract significantly reduced survival of young adult worms to 57% [107]

*Amaranthaceae*					
(3)	Ethanolic extract	Leaves of *Amaranthus spinosus* L.	West Africa	In vivo FEC assay against *Ascaris suum*, *Hyostrongylus rubidus* and *Trichuris trichiur*a in pigs	More than 80% reduction of FEC on day 7 posttreatment with 0.5 g/kg [125]
(4)	Ethanolic extract			In vitro activity against GIN from goats	60% mortality at 100 mg/mL [126]
(5)	Aqueous and methanolic extracts	Whole plant of *Chenopodium album* L.	East Africa	In vitro antimotility, and EHIA against *H*. *contortus* and in vivo against FEC in mixed parasite infected sheep	LC_50_ = 0.134 mg/mL against egg hatching and significantly reduced FEC (93.9% at 3.0 g/kg on day 13 posttreatment [127]

*Anacardiaceae*					
(6)	Acetone extract and fractions	Leaves of *Anacardium occidentale* L.	West Tropical Africa	In vitro EHIA and larval development and viability assays against *H*. *contortus*	LC_50_ of acetone extract was 0.31 and 1.72 mg/ml for hatchability and larval viability test, respectively [122]
(7)	Ethanolic extracts	*Mangifera indica* L.	Tropical Africa	In vitro activity against GIN from goats	Extract induced 50% mortality at 100 mg/mL [126]
(8)	Aqueous extract	Leaves, stem, and root barks of *Spondias mombin* L.	Tropical Africa	Paralysis and death time in vitro assay against earthworm *E*. *eugeniae*	Leaf extract caused paralysis and death at 15 ± 0.33 and 34 ± 0.65 min, respectively [128]
(9)	*n*-Hexane, acetone, and aqueous extracts	Leaves of *Spondias mombin* L.		In vitro activity against adult *Haemonchus placei*	All extracts show some level of activity against the worms with LC_50_ values of 104, 30.5 and 56.27 mg/mL, respectively [129]

*Annonaceae*					
(10)	Aqueous extract	Leaves of *Annona muricata* L.	West Africa	In vitro EHIA, and larval mortality assay against *H*. *contortus*	Effective against egg hatching, *L*3 larvae (84.91%) and adult worms and 89.08% inhibition at 33% v/v [130]
(11)	Aqueous extracts	Stem bark of *Annona senegalensis* Pers.	Tropical Africa	In vitro faecal egg hatch inhibition activity against *H*. *contortus* in sheep	Concentration-dependent decline in larval recovery (88.5 ± 3.1% at 7.1 mg/ml) [131]
(12)	Aqueous extract	Seeds of *Monodora tenuifolia* Benth.	West and East Africa	In vitro EHIA against mixed intestinal nematodes of goats	Significant reduction in egg hatching (93%) at 100 mg/ml [87]
(13)	Ethanolic extract	Leaves and stem bark of *Polyalthia longifolia* (Sonn.)	West Africa	In vitro larval mortality and in vivo FEC assays against H. bakeri in mice	Both extracts possess inhibitory effects on larvae and FEC. At 400 mg/kg the extracts reduced FEC to 0.60 ± 0.24 and 0.40 ± 0.24 EPG, respectively [132]
(14)	Methanolic extracts	Seeds and leaves of *Xylopia aethiopica* A. Rich	Tropical Africa	In vivo assay of postmortem worm recovery against *Nippostrongylus brasiliensis* in rats	Extract produced about 76% deparasitisation [133]
(15)	Ethanolic extracts	Fruits and leaves of *Xylopia aethiopica* A. Rich.		In vitro activity against *P*. *posthuma*	At 30 mg/ml, the fruit extract caused paralysis and death in 65.34 ± 7.05 and 81.72 ± 19.63 min, respectively, whilst leaf extract caused same effects in 69.27 ± 0.00 and 96.39 ± 0.00 min, respectively [134]

*Apocynaceae*					
(16)	Hydroethanolic extract	Stem and root barks of *Alstonia boonei* De Wild	Tropical West and East Africa	In vitro assay against *P*. *Posthuma*	Both extracts induced significant paralysis (17.00 ± 2.10 and 93.00 ± 2.04 min) and death (100.00 ± 2.47 and 151.00 ± 2.27 min), respectively [135]
(17)	Aqueous and ethanolic extracts	Stem bark of *Alstonia boonei* De Wild		In vitro paralysis and mortality assay against earth worms-*Lumbricus terretris*	Both extracts induced paralysis (43.50 ± 7.67 and 34.89 ± 2.48 min, respectively) [99]
(18)	Crude powder, aqueous and methanolic extracts	Flowers of *Calotropis procera* Ait. F.	Tropical Africa	In vitro assay against adult motility, and in vivo FEC against *H*. *contortus* in sheep	Powder and aqueous extract reduced FEC (88.4 and 77.8% at 3 g/kg on day 7 and 10 posttreatment, respectively). Aqueous extract induced 70% worm paralysis at 6 h of 25 mg/ml treatment [121]
(19)	Dried and fresh latex	Latex of *Calotropis procera* Ait. F.		In vitro activity against adult *P*. *posthuma*	Latex possess wormicidal activity (100% mortality at 60 min of 100 mg/ml treatment) and caused causing irreversible paralysis in lower doses [136]
(20)	Methanolic extracts	Stem bark and leaves of *Rauvolfia vomitoria* Afzel.	Tropical Africa	In vitro activity against *P*. *posthuma*	At 50 mg/ml, the two extracts caused paralysis at 11.17 ± 0.08 and 21.68 ± 0.10 min and death at 21.67 ± 0.73 and 143.35 ± 1.41 min, respectively [137]
(21)	Methanolic extracts	Stem bark and leaves of V*oacanga africana* Stapf ex. Scott-Elliot	Tropical Africa	In vitro activity against *P*. *posthuma*	At 50 mg/ml, both extracts caused paralysis at 7.03 ± 0.49 and 22.55 ± 0.57 min and death at 14.77 ± 0.12 and 113.99 ± 1.01 min, respectively [137]

*Asparagaceae*					
(22)	Aqueous extract	Sisal waste liquid of *Agave sisalana* Perrine ex Engelm.	West Tropical Africa	In vivo FEC assay, coprocultures and postmortem worm counts in goats	Extracts reduced FEC (50.3%) and faecal worm count (80%) at 1.7 g/kg with no apparent toxicity to the animals [119]
(23)	Aqueous extracts	Leaf, scape, and bulb of *Drimia indica* (Roxb.) Jessop.	East and West Africa	In vitro action against adult *P*. *posthuma*	All extracts had significant anthelmintic effects. The hot aqueous extract of the leaf was the most active, inducing paralysis at 41.3 ± 0.94 min and death at 50 ± 0.81 at 5 mg/ml [104]

*Asteraceae*					
(24)	Saponin fraction	Aerial parts of *Achillea wilhelmsii* K. Koch	North Africa	In vitro assay on *P*. *posthuma*, tapeworms (*R*. *spiralis*), and adult roundworms (*A*. *galli*)	At 40 mg/ml, *A*. *wilhelmsi* fraction was 1.96 and 2.12 times more potent than albendazole against *P*. *posthuma* and *R*. *spiralis*, respectively [65]
(25)	Aqueous extract	Leafy stems of *Ageratum conyzoides* L.	Tropical Africa	In vitro effects on adult *L*. *terrestris*	Extract caused 100% mortality at 62 ± 0.28 min [138]
(26)	Aqueous and ethanolic extracts	Aerial parts of *Artemisia absinthium* L.	North Africa	In vitro worm motility assay against *H*. *contortus* and in vivo FEC in sheep nematodes	Both extracts were active against ovine nematodes. Ethanolic extract caused FECR of 90.46% at 2.0 g/kg on day 15 posttreatment, and >80% inhibition of worm motility in vitro after 8 h with 25 mg/ml treatment [139]
(27)	Aqueous and methanolic extracts	Whole plant of *Artemisia brevifolia* Wall.	South and Tropical Africa	In vitro inhibition of adult worm motility against H. *contortus* and in vivo FEC assay against *H*. *contortus*, *T*. *colubriformis*, *T*. *axei*, *O*. *columbianum*, *S*. *Papillosus* and *T*. *ovis* in sheep	Methanolic extract inhibited motility (80% at 6 h with 25 mg/ml treatment) and aqueous extract reduced FEC in vivo (67.2% at 3 g/kg on day 14) [140]
(28)	Aqueous and ethanolic extracts	Leaves of *Vernonia amygdalina* Del.	Tropical Africa	In vitro paralysis and mortality assay against earth worms-*Lumbricus terretris*	Both extracts caused significant paralysis (59.94 ± 8.25 and 33.18 ± 12.41 min, respectively) [99]
(29)	Aqueous extract			In vitro faecal egg hatch inhibition activity against *H*. *contortus* in sheep	Extract had no significant inhibition at 11.2 mg/ml [131]
(30)	Acetone extract			In vitro testing on eggs, infective larvae, and adult stages of *H*. *contortus*	Extract was active with 42% ovicidal effect, 70% inhibition of larval migration 90% adulticidal effects at 300 *μ*g/mL [141]
(31)	Chloroformic and ethanolic extracts	Stem of *Vernonia amygdalina* Del.		In vitro activity against adult *P*. *posthuma*	Chloroformic extract was more active causing paralysis and death at 11.95 ± 0.28 and 41.74 ± 2.21 min, respectively, at 75 mg/ml [142]

*Burseraceae*					
(32)	Aqueous extracts	Leaves, stem, and root barks of *Commiphora africana* A. Rich, Engl.	Tropical Africa	Paralysis and death time in vitro assay against earthworm *E*. *eugeniae*	Leaf extract induced paralysis and death at 17 ± 0.72 and 87 ± 6.89 min, respectively [128]
(33)	Aqueous and methanolic extracts	Stem bark of *Boswellia dalzielii* Hutch.	West Africa	In vitro assay against egg hatching of *C*. *elegans* strains	Both extracts, respectively, yield 53.8% and 69.1% egg hatch inhibition at 2 mg/ml [106]

*Combretaceae*					
(34)	Aqueous extract	Leaves of *Anogeissus leiocarpus* (DC.) Guill. & Perr.	West, Central and East Africa	In vivo effects on FEC in sheep naturally infected with gastrointestinal nematodes	Dose-dependent FECR and worm burden reduction (39.5% and 33%, respectively, after 3 consecutive days 400 mg/kg treatment) [143]
(35)	Aqueous decoction			In vitro EHIA, larvicidal assay and mortality of adult *H*. *contortus*	Significant ovicidal (ED_50_ = 409.5 *μ*g/mL), and larvicidal (100% eclodibility inhibition at 1.2 mg/ml) actions. Active against adult worms, but not dose dependent [89]
(36)	Acetone extract and fractions			In vitro EHIA and larval viability assays against *H*. *contortus*	The extract and fractions exhibited concentration-dependent ovicidal and larvicidal activity. LC_50_ of 360 and 509 *μ*g/ml, respectively, for the acetone extract [92]
(37)	Aqueous and ethanolic extracts	Leaves and bark of *Anogeissus leocarpus* (DC.) Guill. & Perr.		In vitro activity against *C*. *elegans* (wild type) larvae	Ethanolic extract of the bark was the most active with LC_50_ of 380 *μ*g/ml [144]
(38)	Methanolic and chloromethylenic extracts	Leaves, roots, and bark of *Anogeissus leocarpus* (DC.) Guill. & Perr.		In vitro activity against *Rhabditis pseudoelongata*	All extracts possess anthelmintic action with EC_50_ between 2.5 *μ*g/ml and 10 *μ*g/ml [145]
(39)	Fractions of ethanolic extract	Stem bark of *Anogeissus schimperi* Hochst.	Tropical Africa	In vivo activity against *Nippostrongylus braziliensis* in rats.	Aqueous fraction showed 64.15% efficacy at 50 mg/kg body weight [146]
(40)	Acetone extract and fractions	Leaves of *Combretum molle* R. Br. ex G. Don	Tropical East Africa	In vitro EHIA, larval development, and viability assay in *H*. *contortus*	Extract and fractions exhibited ovicidal (LC_50_ = 0.87 mg/ml) and larvicidal (LC_50_ = 0.60 mg/ml) action against *H*. *contortus* [147]
(41)	Ethanolic extracts	Leaves of *Combretum mucronatum* Schumach. & Thonn.	Tropical Africa	In vitro adulticidal and larvicidal action against *A*. *ceylanicum*, *H*. *bakeri*, and *T*. *muris* and in vivo against these parasites in NMRI mice	Extract possess nematicidal activities with in vitro minimum lethal concentration of 10 *μ*g/mL against *T*. *muris* and a worm burden reduction of 85.3% in vivo [148]
(42)	Hydroethanolic extract			In vitro action against *C*. *elegans* larvae	Extract caused 41.9% inhibition of larvae at 1 mg/ml [149]
(43)	Hydroethanolic extract and fractions			In vitro action against *C*. *elegans* L4 larvae.	Extract exhibited moderate activity with LC_50_ of 1.67 mg/ml. Ethyl acetate fraction was active with LC_50_ of 1.73 mg/ml [150]
				Atomic force microscopy of *C*. *elegans* treated with tannin-enriched extract.	Ultrastructural changes were reported in the cuticle but no morphological changes on the intestines [151]
(44)	Aqueous and methanolic extracts	Stem bark *Guiera senegalensis* J. F. Gmel.	Tropical Africa	In vitro EHIA and larval development of *C*. *elegans* (wildtype and ivermectin-resistant DA1316) strains	Methanolic extract inhibited egg hatch (>80%) and larval development (>90%) in both strains [152]
(45)	Pet. ether, DCM, ethyl acetate, methanolic and aqueous extracts	Dry fallen leaves of *Terminalia catappa* L.	Tropical West Africa	In vitro EHIA and larval mortality assay against *H*. *contortus*	98.9% inhibition of egg hatching and 98.9% larval reduction by DCM extract at 6.25 mg/ml [153]
(46)	Methanolic and chloromethylenic extracts	Leaves of *Terminalia glaucescens* Planch. ex Benth.	Tropical Africa	In vitro activity against *Rhabditis pseudoelongata*	Both extracts with active with EC_50_ of 2.5 *μ*g/ml each [145]

*Cucurbitaceae*					
(47)	Aqueous and methanolic extracts	Seeds of *Citrullus lanatus* (Thunb.) Mansf.	East and South Africa	In vitro paralysis and mortality effects against *L*. *terrestris*	Aqueous extract at 50 mg/ml induced paralysis and death at 38.49 ± 1.20 and 58.2 ± 3.41 min, respectively [154]
(48)	Aqueous and methanolic extracts	Seeds of *Cucurbita pepo* L.	West Africa	In vitro paralysis and mortality effects against *L*. *terrestris*	Aqueous extract caused concentration-dependent paralysis and death of the worms (37.25 ± 1.60 and 50.49 ± 2.28 min, respectively) at 50 mg/ml [154].
(49)	Ethanolic extracts	Leaves of *Momordica charantia* L.	Tropical Africa	In vitro action against adult *C*. *elegans* (wild type, *N*2, Bristol)	Extracts of plants from different ecological zones showed varying degree of activities with LC_50_ between 473 and 997 *μ*g/ml [155].
(50)	Ethanolic extracts	Fruits of *Momordica charantia* L.		In vitro activity against GIN and in vivo FECR assay in goats	100% mortality at 100 mg/ml and 78% FECR on day 9 posttreatment [126]
(51)	Aqueous and methanolic extracts	Seeds of *Telfairia occidentalis* Hook. f.	Tropical Africa	In vitro paralysis and mortality effects against *L*. *terrestris*	Aqueous extract at 50 mg/ml caused paralysis and death in 42.97 ± 1.45 and 66.63 ± 4.10 min, respectively [154]

*Euphorbiaceae*					
(52)	Petroleum ether, chloroform, and methanol extracts	Leaves of *Alchornea cordifolia* (Schumach.) Müll. Arg.	Tropical Africa	In vitro activity against *E*. *eugeniae* (earthworms)	The methanol extract was the most active inducing paralysis and death at 26.28 ± 0.575 and 57.30 ± 0.370 min, respectively, at 12 mg/ml concentration [156]
(53)	Methanolic and chloromethylenic extracts			In vitro activity against *Rhabditis pseudoelongata*	The chloromethylenic extract and alkaloidal fraction significantly inhibited the worms with EC_50_ of 2.5 *μ*g/ml [145]
(54)	Aqueous and ethanolic extracts	Leaves of *Euphorbia hirta* L.	Tropical Africa	In vitro activity against *C*. *elegans* (wild type) larvae	Ethanolic extract was the most active with LC_50_ of 2.0 mg/ml [144]
(55)	Methanolic and chloromethylenic extracts	Leaves of *Mallotus oppositifolius* Müll. Arg.	West Africa	In vitro activity against *Rhabditis pseudoelongata*	Both extracts and alkaloid fraction were active with EC_50_ of 2.5 *μ*g/ml against the worms [145]

*Fabaceae*					
(56)	Methanolic extract	Fruits of *Acacia nilotica* (L.) Del.	Tropical Africa	In vitro EHIA, adult motility, and larval development assay against *H*. *contortus* and in vivo against *H*. *contortus*, *T*. *circumcincta*, and *T*. *ovis* in sheep.	LC_50_ = 512.86 and 194.98 *μ*g/mL against egg hatch and larval development, respectively. FEC reduced by 78.5% on day 13 post 3.0 g/kg treatment [97]
(57)	Hydroalcoholic extracts			In vitro larvicidal action against *C*. *elegans* strains and *Onchocerca ochengi*	LC_50_ = 10.8 ± 0.3 *μ*g/mL against microfilariae of *O*. *onchengi* and 350 ± 1.1 *μ*g/mL against *C*. *elegans* WT [110]
(58)	Dry feed (browse meal)	Leaves of *Acacia polyacantha* Wild.	Ethiopia, South Africa	In vivo effect on FEC and worm burdens in experimentally infected sheep and goats	Moderate reduction in FEC and worm burden: 27% and 13%, respectively, in sheep and 19% FEC reduction in goats [112]
(59)	Acetone extracts of indigenous browses	Leaves of *Acacia senegal* (L.) Willd.	Tropical Africa	In vitro EHIA, LMIA and adult motility inhibition assays (AMIA) against *H*. *contortus*	Effective against egg hatching (38% at 300 *μ*g/mL), with no dose-dependent inhibition of larvae and adults [141]
(60)	Acetone extracts of indigenous browses	Leaves of *Acacia seyal* Del.	Tropical Africa	In vitro EHIA, LMIA and AMIA against *H*. *contortus*	Concentration-dependent ovicidal effects (34% at 300 *μ*g/mL) with minimal effects on larvae and adult worms [141]
(61)	Acetone extracts of indigenous browses	Leaves of *Acacia tortilis* (Forssk.) Hayne	Tropical Africa	In vitro EHIA, LMIA and AMIA against *H*. *contortus*	Concentration-dependent ovicidal effects (39% at 300 *μ*g/mL) but no significant larvicidal and adulticidal activity [141]
(62)	Methanolic and chloromethylenic extracts	Leaves of *Afrormosia laxiflora* (Benth. ex Baker) Harms	West Africa	In vitro activity against *Rhabditis pseudoelongata*	Both extracts and alkaloid fraction were active with EC_50_ of 2.5 *μ*g/ml against the worms [145]
(63)	Ethanolic extract	Stem bark of *Afzelia africana* Sm.	West, Central and East Africa	In vitro EHIA against *H*. *contortus*	About 90.9% egg hatch inhibition at 5 mg/ml [88]
(64)	Aqueous, methanolic, and chloroformic extracts	Root bark of *Albizia anthelmintica* Brongn.	Southern Africa	In vitro EHIA and larvicidal activity against strongyle-type sheep nematode	The aqueous extract inhibited egg hatching, larval development, and survival of worms (ED_50_ = 144.2, 65.2 and 312.4 *μ*g/mL, respectively). The methanolic extract only caused high mortality of larvae (ED_50_ = 11.8 *μ*g/mL). The chloroformic extract had moderate effects on larval development (ED_50_ = 208.0 *μ*g/mL) [86]
(65)	Aqueous extract			In vivo efficacy against *Haemonchus* spp., *Trichostrogylus* spp. and *Oesophagostomum* spp. mixed infection in sheep	Efficacy of 89.8% was observed against the nematodes [157]
(66)	Aqueous and ethanolic extract	Stem bark of *Albizia schimperiana* Oliv.	Tropical Africa	In vitro ovicidal and larvicidal activity against *H*. *contortus*	Ovicidal activities with ED_50_ = 0.11 *μ*g/mL of aqueous extract and significantly inhibited larval development (99.31% at 50 mg/ml) [158]
(67)	Methanolic extract	Seeds, leaves, flowers of *Butea monosperma* (Lam.) Kuntze	West Tropical Africa	In vitro assay against *C*. *elegans* (wild type) larvae	Extract of seeds very active against *C*. *elegans* (ED_50_ = 901.5 *μ*g/mL) in microwell assay [109]
(68)	Aqueous and methanolic extracts	Seed kernel of *Caesalpinia crista* L.	Tropical Africa	In vitro antimotility, and EHIA against *H*. *contortus* and in vivo against FEC in mixed parasite infected sheep	LC_50_ = 0.449 mg/ml in egg hatch test. EPG reduced by 82.2% at dose of 3.0 g/kg on day 5 posttreatment [127]
(69)	Aqueous decoction	Stem bark of *Daniellia oliveri* (Rolfe) Hutch. & Dalz.	Sudan, Senegal, Egypt, Uganda	In vitro EHIA, larvicidal assay and mortality of adult *H*. *contortus*	Ovicidal (ED_50_ = 245.9 *μ*g/mL and larvicidal (100% eclodibility inhibition at 1.2 mg/ml) actions. Active against adult worms, but not dose-dependent [89]
70)	Acetone extracts of indigenous browses	Leaves of *Millettia ferruginea* (Hochst.) ex. Baker	Tropical Africa	In vitro testing on eggs, infective larvae, and adult stages of *H*. *contortus*	Ovicidal action (35% at 300 *μ*g/mL) but no significant effects against larvae and adult worms [141]
(71)	Aqueous methanolic extract	Leaves of *Mimosa pudica* L.	East Africa	In vitro egg hatch assay and in vivo using *H*. *bakeri* experimentally infected adult albino mice	Extract significantly inhibited egg hatch in vitro (LC_50_ of 1.160 *μ*g/mL) and reduced worm count in vivo [118]
(72)	Aqueous and ethanolic extract	Leaves of *Senna occidentalis* (L.) Link	Tropical Africa	In vitro ovicidal and larvicidal activity against *H*. *contortus*	ED_50_ = 0.13 *μ*g/mL against egg hatch and 96.36% inhibition of larval development [158]
(73)	Chloroformic and methanolic extracts	Aerial parts of *Tephrosia spinosa* (L.f.) Pers.	Tropical Africa	In vitro anthelmintic activity on adult Indian earth worms (*Pheretima posthuma*)	Significant paralytic effect on worms. Chloroformic extract (14.34 ± 0.04 min), methanolic extract (21.98 ± 0.15 min) [98]
(74)	Aqueous extracts	Leaves of *Tephrosia villosa* (L.) Pers.	West Africa	In vitro EHIA and larval development assays against *H*. *contortus*	93.2 ± 0.9% inhibition of egg hatch and 81.8 ± 2.99% inhibition of larval development at 500 mg/ml [94]
(75)	Aqueous extracts	Leaves of *Tephrosia vogelii* Hook.f.	Tropical Africa	In vitro EHIA and larval development assays against *H*. *contortus*	Significant inhibition against of egg hatch (95.8 ± 1.71%) and larval development (99.0 ± 1.41%) at 500 mg/ml [94]

*Lamiaceae*					
(76)	Aqueous and ethanolic extract	Flowers and roots of *Leonotis ocymifolia* (Burm.f.) Iwarsson	Southern and East Africa	In vitro ovicidal and larvicidal activity against *H*. *contortus*	Aqueous extract had ED_50_ = 0.25* μ*g/mL against egg hatch and 100% larval development inhibition at 50 mg/ml [158]
(77)	Aqueous and ethanolic extract	Aerial parts of *Leucas martinicensis* (Jacq) R.Br.	Tropical Africa	In vitro ovicidal and larvicidal activity against *H*. *contortus*	ED_50_ = 0.09* μ*g/mL of aqueous extract against egg hatch and inhibited larval development by 99.85% at 50 mg/ml [158]
(78)	Hexane and ethanolic extracts	Fruits of *Ocimum basilicum* L.	West Tropical Africa	In vitro activity against the earthworm *E*. *eugeniae*	Ethanolic extract was most active causing paralysis in 11.85 ± 0.71 min and death in 24.74 ± 0.42 min at 5 mg/ml. This was significantly active than mebendazole, the positive control [159]
(79)	Acetone extract	Leaves of *Ocimum gratissimum* Forssk.	Tropical Africa	In vitro activity against adult *H*. *placei*	Concentration-dependent inhibition of parasites with LC_50_ of 17.70 mg/ml [160]
(80)	Essential oils	Whole plant of *Ocimum sanctum* L.	Across Africa	In vitro assay against *C*. *elegans* (wild type) larvae	Oil and eugenol (ED_50_ of 62.1* μ*g/mL) showed activity against *C*. *elegans* larvae [108]
(81)	Essential oil	Aerial parts of *Thymus bovei* Benth.	North Africa	In vitro wormicidal action on adult *P*. *posthuma*	At 10 mg/ml, paralysis and death time were 19.61 ± 0.88 and 47.32 ± 0.94 min, respectively [161]
(82)	Aqueous and methanolic extracts	Stem barks of *Vitex doniana* Sweet	Tropical Africa	In vitro assay against egg hatching of *C*. *elegans* strains	At 2 mg/ml, both extracts had 83.8% and 92.3% ovicidal activity, respectively, against the ivermectin-resistant strain DA1316 [106]
(83)	Saponin fraction	Aerial parts of *Teucrium stocksianum* Boiss.	Tropical East and North Africa	In vitro assay on *P*. *posthuma*, tapeworms (*R*. *spiralis*), and adult roundworms (*A*. *galli*)	At 40 mg/ml, the fraction was 1.89, 1.96 and 1.37 times active than albendazole against the three organisms, respectively [65]

*Meliaceae*					
(84)	Aqueous, methanolic extract and fraction	Seeds of *Azadirachta indica* A. Juss.	Tropical Africa	In vitro EHIA and larval survival assay against *H*. *contortus*	All fractions exhibited dose-dependent effects. Ethyl acetate fraction had LC_50_ = 21.32* μ*g/ml against egg hatching and 83% wormicidal effects at 50 mg/ml 1 h postexposure [162]
(85)	Aqueous and methanolic extracts			In vivo inhibition of FEC and larval counts in sheep naturally infected with *H*. *contortus* and *Trichostrongyus* spp	Both extracts induced 29.3% and 40.2% reduction in EPG on day 15 posttreatment with 3 g/kg [163]
(86)	Aqueous extract	Leaves of *Azadirachta indica* A. Juss.		In vitro activity against *P*. *posthuma*, tapeworms (*Raillietina spiralis*) and roundworms (*Ascaridia galli*)	The extract significantly reduced paralysis (17 ± 0.32, 13 ± 0.85, 19 ± 0.50 min) and death time (30 ± 0.11, 38 ± 1.20, 40 ± 0.50 min), respectively, against all parasites [164]
(87)	Crude feed			In vivo efficacy against bovine strongylosis	Induced about 98% FECR on day 14 posttreatment [165]
(88)	Diethyl etheric, chloroformic, ethanolic and methanolic extracts			In vitro activity against microfilariae of *Setaria cervi*	Methanolic and ethanolic extracts were the most active with 87% and 60% mortality at 45 min posttreatment with 200* μ*g/ml [166]
(89)	Traditional preparations			In vivo activity (FEC and TWC) against *H*. *polygyrus* in mice	26% FECR and 15% TWCR at 7 days posttreatment [84]
(90)	Ethanolic extracts			In vitro activity against GIN and in vivo FECR assay in goats	100% mortality at 100 mg/ml with 81% reduction in EPG on day 9 posttreatment [126]
(91)	Aqueous extracts	Leaves, stem, and root barks of *Azadirachta indica* A. Juss.		In vitro EHIA and larval inhibition against strongylid nematodes of small ruminants	Dose-dependent activity with over 90% inhibition of survival and about 50% inhibition of egg hatch at 100 mg/ml [167]
(92)	Aqueous and ethanolic extracts	Stem bark *Khaya senegalensis* A. Juss.	West, Central and East Africa	In vitro activity against larvae of strongyles, and in vivo against mixed intestinal nematodes in sheep	Both extracts are active with no significant difference between them. Ethanolic extract had LC_50_ of 0.51 mg/ml and 88.82% FECR at 500 mg/kg, respectively [168].
(93)	Aqueous and ethanolic extracts	Leaves and bark of *Khaya senegalensis* A. Juss.		In vitro activity against *C*. *elegans* (wild type) larvae	Ethanolic extract of the bark and leaves had LC_50_ of 470* μ*g/ml and 1.0 mg/ml, respectively, against *C*. *elegans* [144].
(94)	1 : 1 DCM: methanolic extract	Whole plant of *Lansium domesticum* Corrêa	Tropical East Africa	In vitro activity against L3, L4 and adult *C*. *elegans* (wild type, Bristol N2)	Extract significantly reduced survival of adult worms to 59% [107].

*Musaceae*					
(95)	Dry feed	Leaves of *Musa* spp	Tropical Africa	In vivo assay against *H*. *contortus*, and *T*. *colubriformis* in sheep	Significant inhibition of egg hatching in *T*. *colubriformis* (91% on day 15 posttreatment) [169]
(96)	Aqueous extracts	Leaves, pseudostems, and hearts of *Musa* spp.	Tropical Africa	In vitro activity against larval development in *H*. *contortus*	Significant inhibition of larval development (>96.9%) recorded at 75 mg/ml [170]
(97)	Aqueous extracts	Leaves, pseudostems, and heart of *Musa* spp. cv. Prata anã	Tropical Africa	In vitro egg hatch, and larval development assays, and in vivo FEC reduction assay against *H*. *contortus* in lambs	The leaf extract exhibited ovicidal (LC_50_ = 0.19 mg/ml) and larvicidal activities in vitro, and 33.0% FECR after 1 week of treatment with 303 mg/kg body weight [171]
(98)	Dry feed	Leaves of *Musa* x *paradisiaca* L.	Tropical Africa	In vivo assay as feed for lambs experimentally infected with *H*. *contortus*	Significant FECR after 21 consecutive days of feeding with 7000 g/lamb/day [172]
(99)	Hydromethanolic extracts			In vitro activity against *H*. *contortus* eggs, adult motility assay in vivo effect on FEC and larval counts in sheep naturally infected with mixed species of nematodes	Extract exhibited strong in vitro activity on egg hatching (LC_50_ = 2.13* μ*g/mL) and caused 80.7% reduction in FEC in vivo at 8 g/kg body [173]
(100)	Methanolic extracts	Roots of *Musa* spp. (*M*. *paradisiaca*, *M*. *sapientum*, and *M*. *nana*)	Tropical Africa	In vitro assay against sheep tapeworm (*Moniezia benedeni*), roundworm (*Ascaris lumbricoides*), and adult earthworm (*Esenia fetida*)	All extracts showed significantly paralytic effects on the worms. *M*. *paradisiaca* was more active with 26.07 ± 1.7, 57.08 ± 1.32, and 80.04 ± 0.5 min paralysis time against the three worms, respectively [173]

*Phyllanthaceae*					
(101)	Methanolic and chloromethylenic extracts	Leaves and bark of *Bridelia ferruginea* Benth.	Tropical Africa	In vitro activity against *Rhabditis pseudoelongata*	The dichloromethane extracts were more active than the methanolic extracts with EC_50_ of 2.5* μ*g/ml and 5* μ*g/ml, respectively [145]
(102)	Hydroethanolic extract	Shoots of *Phyllanthus urinaria* L.	Tropical Africa	In vitro action against *C*. *elegans* larvae	Extract caused 10.8% inhibition of larvae (89.2% survival) at 1 mg/ml [149]

*Piperaceae*					
(103)	Aqueous extract	Seeds of *Piper guineense* Thonn.	West, East, And Central Africa	In vitro EHIA against mixed intestinal nematodes of goats	90% inhibition of egg hatch at 100 mg/ml [87]
((104)				In vitro effects on adult *L*. *terrestris*	Extract induced 100% mortality of worms at 40 ± 0.68 min [138]
((105)	Ethanolic extracts	Seeds of *Piper betle* L.	Tropical East Africa	In vitro activity against GIN from goats	70% mortality at 100 mg/ml [126]

*Poaceae*					
(106)	Acetone extract	Leaves of *Cymbopogon citratus* Stapf.	Tropical Africa	In vitro activity against adult *H*. *placei*	Extract was active against the worm with LC_50_ of 56.04 mg/ml [160]
(107)	Ethanolic extract	*Cynodon dactylon* (L.) Pers.	Tropical East Africa	In vitro activity against GIN from goats	Extract exhibited 50% mortality at 100 mg/ml [126]

*Primulaceae*					
(108)	Aqueous and ethanolic extracts	Seeds of *Embelia rowlandii* Gilg	Tropical West Africa	In vitro ovicidal and larvicidal activities of extracts against *H*. *bakeri*	The two extracts showed concentration-dependent ovicidal (82.5 and 46.9%), hatch inhibition (85.8 and 41.0%), and *L*1 larvicidal (86.0 and 61.2%) effects, respectively, at 5 mg/ml [174]
(109)	Aqueous extract	Fruits and leaves of *Myrsine africana* L.	East and South Africa	In vivo efficacy against *Haemonchus* spp., *Trichostrogylus* spp., and *Oesophagostomum* spp. mixed infection in sheep	Extract exhibited 77% efficacy against the parasites [157]
(110)	Traditional preparations	Seeds of *Myrsine africana* L.		In vivo activity (FEC and TWC) against *H*. *polygyrus* in mice	16% FECR and 10% TWCR at 7 days posttreatment [84]
(111)	Feed paste	Fruits of *Rapanea melanophloeos* L.	Tropical Africa	In vivo activity against *H*. *polygyrus* in mice	Moderate reduction in FEC and with no apparent effects on total worm counts [84]

*Rosaceae*					
(112)	Serial ether, chloroformic, methanolic, and chloromethylenic extracts	Stem bark of *Hagenia abyssinica* Bruce J.F. Gmel	Ethiopia, Malawi, Tanzania, DRC	In vitro activity against *Panagrellus redivivus* and *C*. *elegans*	Relatively polar extracts possess anthelmintic activities against the test organisms. Methanolic extract at 20 mg/ml caused 67% mortality in *C*. *elegans* after 24 h [105]
(113)	Methanolic extract	Fruits of *Rubus fruticosus* Marshall	North Africa	In vitro activity against *R*. *spiralis* and *A*. *galli*	89.83% and 84.2% parasiticidal activity at 40 mg/ml against *R*. *spiralis* and *A*. *galli*, respectively [175]

*Rubiaceae*					
(114)	Ethanolic, aqueous extracts	Stem bark of *Canthium mannii* Hiern.	West Africa	In vitro EHIA against *Ancylostoma caninum*	Ethanolic extract caused 90% egg hatch inhibition at 1 mg/ml after 48 h. The aqueous extracts had <50% eclodibility inhibition [96]
(115)	Methanolic and chloromethylenic extracts	Leaves and roots of *Morinda lucida* A. Gray	Tropical Africa	In vitro activity against *R*. *pseudoelongata*	Both extracts were active with EC_50_ of 2.5* μ*g/ml against the worms [145]
(116)	Hydroethanolic extract	Stem bark of *Zanthoxylum zanthoxyloides* A. Gray		In vitro action against *P*. *posthuma*	Extract induced dose-dependent inhibition with paralysis and death time of 18.17 ± 0.03 and 24.34 ± 0.21 min, respectively, at 50 mg/ml [176]
(117)	Ethanolic extract	Leaves of *Morinda lucida* A. Gray		In vitro effects on egg hatching, infective larvae, and adult worms of *T*. *colubriformis*	Concentration-dependent ovicidal effect, with 18% inhibition of motility at 48 h of 2.5 mg/ml treatment [177]
(118)	Aqueous and ethanolic extracts	Leaves of *Nauclea latifolia* Sm.	West and Central Africa	In vitro EHIA against *H*. *contortus*, *Trichostrongylus* spp., *Strongyloides* spp., *Trichuris ovis* and *Oesophagostomum* spp. and in vivo in sheep naturally infected with ovine nematodes	LC_50_ of 0.704 and 0.650 mg/ml for the aqueous and ethanolic extract in vitro, respectively, for mixed parasites. Both extracts caused reduction of FEC in vivo with improved haemoglobin and leukocytosis in worm-infested sheep [95]
(119)	Aqueous extract	Stem bark of *Nauclea latifolia* Sm.		In vivo efficacy study against strongyle ovine nematodes	FEC was significantly reduced (93.8%) in infected animals treated with 1600 mg/kg body weight for 5 consecutive days [178]

*Rutaceae*					
(120)	Ethanolic extract	Roots of *Clausena anisate* (Willd.) Hook. F.	Tropical Africa	In vitro LMIA against *Ascaris suum*	Extracts were significantly active with EC_50_ of 74* μ*g/mL [24]
(121)	DCM -methanolic (1:1) extract	Root bark of *Teclea trichocarpa* Engl.	East Africa	In vitro EHIA and larval development in *Strongyloides*	Effective against egg hatching (IC_50_ = 185.25* μ*g/mL) and but not larval development at 1 mg/ml [93]
(122)	Ethanolic extract	Root bark of *Zanthoxylum zanthoxyloides* (Lam.) Zepern. & Timler	West Africa	In vitro LMIA against *Ascaris suum*	Extract inhibited larval migration with EC_50_ of 164* μ*g/mL [24]
(123)	Ethanolic extract	Leaves of Zanthoxylum zanthoxyloides (Lam.) Zepern. & Timler		In vitro effects on egg hatching, infective larvae, and adult worms of *Trichostrongylus colubriformis*	Significant inhibition of adult worm motility (87% after 48 of 2.5 mg/ml treatment) [177]

*Zingiberaceae*					
(124)	Methanolic and chloromethylenic extracts	Leaves and rhizomes of *Aframomum sceptrum* (Oliv. & D. Hanb.) K. Schum.	West Tropical Africa	In vitro activity against *Rhabditis pseudoelongata*	Both extract and alkaloid fraction were active with EC_50_ of 2.5* μ*g/ml against the worms [145]
(125)	Aqueous extract	Leafy stems of *Aframomum alboviolaceum* (Ridl.) *K*. *Schum*	Tropical Africa	In vitro effects on adult *L*. *terrestris*	Extract caused 100% mortality at 86 ± 3.21 min [138]
(126)	Crude powder	Rhizomes of *Zingiber officinale* Roscoe	Tropical Africa	In vivo FEC in pigs experimentally infected with *S*. *ransomi*, *H*. *rubidus*, *T*. *axei* and *G*. *urosubulatus*	The powder significantly reduced FEC (92.6% with 25 g/kg treatment against *S*. *ransomi*) [179]
(127)	Aqueous extract and powder			In vivo FEC assay against mixed nematode infection in sheep	Both powder and extract caused a dose-dependent reduction in FEC (25.6% and 66.6%, respectively) [180]

*Others*					
(128)	Hydromethanolic extracts	Whole plant of *Trianthema portulacastrum* L. (*Aizoaceae*)	Tropical Africa	In vitro EHIA, adult motility assay against *H*. *contortus*. In vivo FEC assay in sheep naturally infected with mixed species of nematodes	Extract strongly inhibited egg hatching (LC_50_ = 2.41* μ*g/mL) and significantly reduced FEC (85.6% at 8.0 g/kg on day 15 posttreatment) [181]
(129)	Aqueous and ethanolic extracts	Seeds of *Coriandrum sativum* L. (*Apiaceae*)	Tropical East Africa	In vitro EHIA and effects on adult worms, and in vivo FEC assay against *H*. *contortus* in sheep	Both extracts were active against egg hatching (ED_50_ = 0.12 and 0.18 mg/ml resp.), and moderately reduced FEC (24.5% at 0.9 g/kg) [182]
(130)	Ethanolic extract			In vitro and in vivo FEC assay against *Hymenolepis nana* in mice	Extracts paralysed and killed the worms within 30 min and caused a 100% FECR at 500 mg/kg on day 15 posttreatment [114]
(131)	Aqueous and hydroethanolic extracts	Ripe fruits of *Hedera helix* L. (*Araliaceae*)	North and South Africa	In vitro EHIA, faecal worm burden and adult worm mortality, and in vivo FEC assay against *H*. *contortus* in sheep	Both extracts reduce faecal parasite count (44.2% at 2.25 g/kg) and produced ovicidal effects; ED_50_ = 0.12 and 0.17 mg/ml for aqueous and hydroalcoholic extracts, respectively [183]
(132)	Ethyl acetate extract	Husk of green fruits of *Cocos nucifera* L (*Arecaceae*)	Lowland Tropical Africa	In vitro egg hatching and larval development assays against *H*. *contortus*. In vivo FEC assay in sheep	Extract exhibited larvicidal (99.77% inhibition at 80 mg/ml) and ovicidal (100% inhibition at 5 mg/ml). No statistically significant activity was observed in vivo [184]
(133)	Aqueous acetone extracts	Leaves of *Newbouldia laevis* (*P*. *Beauv*.) Seem. (*Bignoniaceae*)	West Africa	In vitro action against larval exsheathment in *H*. *contortus* and *T*. *colubriformis*	Concentration-dependent inhibition of *H*. *contortus* and *T*. *colubriformis* larval exsheathment (81.65% and 78.6%, respectively) at 600* μ*g/ml [185]
(134)	Ethanolic extract			In vitro effects on egg hatching, infective larvae, and adult worms of *Trichostrongylus colubriformis*	100% inhibition of adult worm motility after 48 of 2.5 mg/ml treatment [177]
(135)	Ethanolic extracts	Leaves of *Ananas comosus* (L.) Merr. (*Bromeliaceae*)	Tropical Africa	In vitro activity against GIN and in vivo FECR assay in goats	100% mortality at 100 mg/ml and 73% reduction in EPG on day 9 posttreatment [126]
(136)	Aqueous extracts	Leaves, stem, and root barks of *Canna bidentata* Bertol. (*Cannaceae*)	Tropical Africa	Paralysis and death time in vitro assay against earthworm *E*. *eugeniae*	Concentration-dependent activity was observed. Root extract caused paralysis and death at 3 ± 0.00 and 5 ± 0.15 min, respectively [128]
(137)	Hydroethanolic extract	Seeds of *Buchholzia coriacea* Engl. (*Capparaceae*)	West and Central Africa	in vitro assay against *L*3 larvae of *Haemonchus contortus* and *Heligmosomoides polygyrus*	Significant larvicidal activity causing 94% and 100% mortality against the two parasites at 100 mg/ml, respectively, and IC_50_ of 16.82 and 11.20 mg/ml, respectively [117]
				In vivo assay in chicken experimentally infected with *Ascardia galli*	Extract had no effect on worm load and FEC in the infected animals [117]
(138)	Latex	Unripped fruits of *Carica papaya* L. (*Caricaceae*)	Tropical Africa	In vivo anthelmintic efficacy against *Ascaridia galli* and *Cappilaria* spp. in poultry	Latex caused 77.7% reduction in FEC after 1 week of treatment [186]
(139)	Aqueous extracts	Seeds of *Carica papaya* L. (*Caricaceae*)		In vivo against GI nematodes in naturally infected goats	Extract exhibited significant reduction in FEC (100%) in 7 days after drenching with 100 mg/ml [187]
(140)				In vivo assay against FEC of *H*. *contortus*, *Trichostrongylus* spp, *Strongyloides* spp, and *Ostertagia* spp. in sheep	Extracts showed 100% reduction in FEC in listed worms after 7 days treatment with 100 mg/ml [188].
(141)				In vivo against FEC in mixed infections in chicks	Improved haematological indices and significantly (100%) reduced FEC in chicks after 2 weeks of treatment with 1 : 10 ml (w/v) extract [116]
(142)				In vivo assay against *H*. *contortus*, *Oesophagostomum* spp., *Trichostrongylus* spp., and *Cooperia* spp. in goats	Improved haematological indices and a significant (100%) FECR 2 weeks after drenching with 1 : 10 ml (w/v) extract) [111]
(143)	Ethanolic extract			In vitro assay against egg hatch, larvae, and adult worms of *T*. *colibriformis*	Significant inhibition of all stages of the worm with 90.5% immobilisation after 48 h treatment at 2.5 mg/ml [177]
(144)	Ethanolic and chlorofomic extracts	Seeds of *Carica papaya* L. (*Caricaceae*)		In vitro activity against adult *P*. *posthuma*	Ethanolic extract was the most active, inducing paralysis and death at 6.69 ± 0.68 and 19.75 ± 0.73 min, respectively, at 75 mg/ml [142]
(145)	Ethanolic and hydroethanolic extracts	Leaves, stem bark, and seeds of *Carica papaya* L. (*Caricaceae*)		Comparative in vitro anthelmintic effect of extracts against adult *P*. *posthuma*	All extracts induced paralysis and caused death of worms, Seeds were more active with 7.21 ± 0.01 and 9.15 ± 0.01 paralysis and death times, respectively, at 5 mg/ml [6]
(146)	Aqueous extracts	Leaves and seeds of *Carica papaya* L. (*Caricaceae*)		In vitro EHIA and larval development assays against *H*. *contortus*	Seeds extract was most active (LD_50_ of 49.94 mg/ml for egg hatch and 49.32 mg/ml against larval development) [94]
(147)	Hydroethanolic extract	Seeds of *Garcinia kola* (Heckel) (*Clusiaceae*)	West and South Africa	In vitro EHIA, and larval mortality assay against *Heligmosomoides bakeri*	18.75% inhibition of the egg-hatch at 100 mg/ml and 76.52% irreversible paralysis of the larvae at 50 mg/ml [91]
(148)	Aqueous extract			In vitro larvicidal effects against strongylid nematodes of goats	Extract exhibited larvicidal action with 53.3%, 66.6% and 73.3% mortality at 50, 100, and 150 mg/ml, respectively [189]
(149)	Hydroethanolic extract	Stem bark of *Garcinia kola* Heckel (*Clusiaceae*)		In vitro action against *P*. *posthuma*	Extract induced dose-dependent paralysis and death of the adult worms at 39.29 ± 0.12 and 54.29 ± 0.01 min, respectively, for 50 mg/ml [176]
(150)	Aqueous extracts	Seeds and stem bark of *Garcinia kola* Heckel (*Clusiaceae*)		In vitro EHIA against strongylid nematodes of small ruminants	Both extracts were ovicidal (98.9% and 100%, respectively) at 100 mg/ml [190]
(151)	Aqueous extract	Root bark of *Hildebrandtia sepalosa* Rendle (*Convolvulaceae*)	Tropical Africa	In vivo efficacy against *Haemonchus* spp., *Trichostrogylus* spp. and *Oesophagostomum* spp. mixed infection in sheep	A 90% reduction in FEC was observed on day 12 posttreatment [157]
(152)	Methanolic extract	Whole plant of *Cyperus difformis* L. (*Cyperaceae*)	Africa	In vitro action against *P*. *posthuma*	The extract showed a concentration-dependent paralysis (66.67 ± 1.8 min) and death (140.7 ± 2.3 min) at 20 mg/ml, respectively. It also improved the activity of albendazole, mebendazole and levamisole when 2 mg/ml of the extract was combined with these drugs [100]
(153)	Ethanolic extract	Leaves of *Diospyros mespiliformis* Hochst. ex A. DC. (*Ebenaceae*)	Tropical Africa	In vivo assay against fecundity of adult *H*. *contortus* in sheep	Extract induced about 55.08% reduction in FEC at 200 mg/kg dose [191]
(154)	Ethanolic extract	Roots of *Anthocleista djalonensis* A. Chev. (*Gentianaceae*)	West Tropical Africa	In vitro against *L*3 larvae of *H*. *polygyrus*	Concentration-dependent lethal effects on *L*3 larvae with LC_50_ of 268.89 mg/ml [192]
(155)	Hydromethanolic extract	Stem bark of *Sacoglottis gabonensis* (Baill.) Urb. (*Humiriaceae*)	West Tropical Africa	In vitro EHIA, and larval mortality assay against *Heligmosomoides bakeri*	100% larval paralysis at 15.63 mg/ml [91]
(156)	1 : 1 DCM: methanolic extract	Whole plant, and leaves of *Picria fel-terrae* Lour. (*Linderniaceae*)	North and East Africa	In vitro effects on various stages of *H*. *contortus*	Extracts showed considerable activity against the parasitic larval stages of *H*. *contortus*. It inhibited (94.1%) the development of *L*3 to *L*4 larvae 7 days posttreatment [193]
(157)				In vitro activity against *L*3, *L*4 and adult *C*. *elegans* (wild type, Bristol N2)	Both extracts significantly affected all 3 stages of *C*. *elegans*. It produced 50% defective egg laying phenotypes [107]
(158)	Aqueous fraction of an ethanolic extract	Whole plant of *Spigelia anthelmia* L. (*Loganiaceae*)	West Africa	In vivo efficacy in *Nippostrongylus braziliensis* infected rats	Efficacy of 74.35 ± 22.29% at 25 mg/kg body weight [194]
(159)	Ethanolic extract	Fruit peels of *Punica granatum* L. (*Lythraceae*)	Ethiopia	In vitro LMIA against *Ascaris suum*	Extracts active against larval migration with EC_50_ of 97* μ*g/mL [24]
(160)	Ethanolic extract	Leaves of *Corchorus olitorius* L. (*Malvaceae*)	Tropical Africa	In vitro activity against GIN from goats	Extract induced 60% mortality at 100 mg/ml [126]
(161)	Chloroformic, pet. ether and ethanolic extracts	Leaves of *Memecylon umbellatum* Burm. (*Melastomataceae*)	Tropical Africa	In vitro activity against adult *Pheretima posthuma*	Ethanolic extract was most potent with paralysis and death time of 29.66 ± 0.66 and 42.33 ± 1.45 min, respectively [195]
(162)	Ethanolic extract	Fruits and seeds of *Sphenocentrum jollyanum* Pierre (*Menispermaceae*)	Tropical Africa	In vitro activity against adult earthworms *Eudrilus eugeniae*, *H*. *placei*, and *T*. *saginata*	Seed extract caused paralysis and death at 18 ± 0.35 and 133 ± 3.75 min, respectively, against *E*. *Eugeniae* [196]
(163)	Feed for snails	Leaves of *Moringa oleifera* Lam. (*Moringaceae*)	West Africa	In vivo against roundworms in snails (*Achatina achatina*)	Significant prevention of parasitisation (*c*^2^ (1) = 14.97; *p*=0.0001) was observed in the treated snails [197]
(164)	Methanolic extract	Stem bark of *Pycnanthus angolensis* (Welw.) Warb. (*Myristicaceae*)	Tropical Africa	In vitro activity against adult earthworms *Eudrilus eugeniae*, *H*. *placei*, and *T*. *saginata*	Extract showed potent activity against all worms studied. At 80 mg/ml, paralysis occurred in the earthworm at 41 ± 1.81 min and death at 67 ± 2.60 min [196]
(165)	Ethanolic extracts	Flower buds of *Syzygium aromaticum* (L.) Merr. & L. M. Perry (*Myrtaceae*)	Tropical Africa	In vitro activity against GIN and in vivo FECR assay in goats	100% mortality at 100 mg/ml and 85% FECR on day 9 posttreatment [126]
(166)	Hydroethanolic and 1:1 DCM: methanolic extracts	Leaves, trunk bark and root bark of *Lophira lanceolata* Tiegh. ex Keay (*Ochnaceae*)	Tropical Africa	In vitro activity against adult *O*. *ochengi* and *L*4 larvae of various strains of *C*. *elegans*	All extracts exhibited 100% inhibition of *O*. *ochengi* at 20* μ*g/mL after 72 h. Ethanolic extract of the leaves was the most active against the drug-resistant strains [68]
(167)	Methanolic and chloromethylenic extracts	Leaves of *Adenia lobata* Engl. (*Passifloraceae*)	Central Africa	In vitro activity against *Rhabditis pseudoelongata*	Both extracts were active with EC_50_ of 5* μ*g/ml each against the worms [145]
(168)	Ethyl acetate and methanloic extracts	Leaves of *Plumbago zeylanica* L. (*Plumbaginaceae*)	Tropical Africa	In vitro effects on paralysis and mortality of *P*. *posthuma*	Ethyl acetate extract was the most active of the two causing paralysis and death at 59.85 ± 3.35 and 80.55 ± 2.66 min, respectively [198]
(169)	Aqueous and ethanolic extract	Leaves and roots of *Rumex abyssinicus* Jacq. (*Polygonaceae*)	East Africa	In vitro ovicidal and larvicidal activity against *H*. *contortus*	ED_50_ = 0.11* μ*g/mL of aqueous extract against egg hatch [158]
(170)	Methanolic extract	Bark of *Ziziphus nummularia* (Burm.f.) Wight & Arn. (*Rhamnaceae*)	North Africa	In vitro EHIA, adult motility, and larval development assay against *H*. *contortus* and in vivo against *H*. *contortus*, *T*. *circumcincta*, and *T*. *ovis* in sheep.	LC_50_ = 676.08 and 398.11* μ*g/ml against egg hatch and larval development, respectively. FECR (84.7%) was recorded on day 13 post 3.0 g/kg treatment [97]
(171)	Hydroethanolic extract	Root bark of *Paullinia pinnata* L. (*Sapindaceae*)	Tropical Africa	In vitro action against *C*. *elegans* larvae	Extract caused 14.8% inhibition of larvae (85.2% survival) [149]
(172)	Hydroethanolic extract			In vitro mortality assay against larvae of *C*. *elegans*, *Toxocara cati*, *Ancylostooma caninum*, and *Trichuris vulpis*. EHIA and LMIA against *H*. *contortus*	Extract was active against some of the test organisms. *C*. *elegans* (LC_50_ of 2.5 mg/ml), *T*. *cati* (LC_50_ of 112* μ*g/mL), and *T*. *vulpis* (LC_50_ = 17* μ*g/mL) [199].
(173)	Aqueous-acetone extract, ethyl acetate, and water partitions			In vitro action against *C*. *elegans* larvae	Ethyl acetate partition was more effective (LC_50_ = 1.1 mg/ml) than the crude extract (LC_50_ = 1.9 mg/ml) and water fraction (LC_50_ = 2.9 mg/ml) [200]
(174)	PAC fractions			In vitro action against *C*. *elegans* larvae	All PAC fractions exhibited a minimum of 70% inhibition against the larvae [200]
(175)	Methanolic and chloromethylenic extracts	Leaves and roots of *Paullinia pinnata* L. (*Sapindacea*e)		In vitro activity against *Rhabditis pseudoelongata*	Extracts of both parts were active against the worm with EC_50_ of 2.5* μ*g/ml [145]
(176)	Aqueous methanolic extract	Aerial parts of *Verbascum thapsus* L. (*Scrophulariaceae*)	North Africa	In vitro activity against *R*. *spiralis* and *A*. *galli*	Extract produced relative paralysis index of 2.08 at 40 mg/ml against *A*. *galli* [201]
(177)	Methanolic extract	Dried fruits of *Brucea javanica* (L.) Merr. (*Simaroubaceae*)	Tropical East Africa	In vivo activity against *Dactylogyrus intermedius* in goldfish	Extract showed profound activity against the parasites (EC_50_ = 49.96 mg/L) [113]
(178)	Aqueous and alcoholic extracts	Leaves of *Nicotiana tabacum* L. (*Solanaceae*)	Tropical Africa	In vitro assay against motility of *Marshallagia marshalli*	Extracts significantly inhibited the motility of the worms, causing 0.44 death rate at 75 mg/ml [77]
(179)	Aqueous and methanolic extracts			In vitro inhibition of motility against *H*. *contortus* and in vivo FEC assay against *H*. *contortus*, *T*. *axei*, *T*. *colubriformis*, *O*. *columbianum*, *S*. *Papillosus* and *T*. *ovis* in sheep	Extracts significantly inhibited worm motility at 6 h postexposure and caused a reduction in FEC (73.6% on day 5 posttreatment with methanolic extract at 3.0 g/kg) [202]
(180)	Ethanolic extracts			In vitro activity against GIN from goats	80% mortality at 100 mg/ml [126]

**Table 2:** Anthelmintic compounds isolated from African medicinal plants.

No.	Compound	Chemical nature/group	Plant source	Reference(s)	Habitats of plant in Africa
(1)	*β*-Asarone	Phenylpropanoid	Rhizomes of *Acorus calamus* L. and *A*. *gramineus* Aiton (*Acoraceae*)	[204]	Southern Africa
(2)	Aspidinol	Phloroglucinol derivative	Leaves of *Leucosidea sericea* Eckl. & Zeyh. (*Rosaceae*)	[205]	Southern Africa
(3)	3-Geranyl-1-(2'-methylbutanoyl)-phloroglucinol	Phloroglucinol derivative	Leaves of *Hypericum roeperianum* Schimp. ex.A.Rich. (*Hypericaceae*)	[206]	West Tropical Africa
(4)	*α*-Bisabolol	Sesquiterpene	Leaves of *Siparuna guianensis* Aubl. (*Siparunaceae*)	[207]	West Africa
(5)	Betulinic acid	Terpenoid	Stem bark of *Berlinia grandiflora* (Vahl) Hutch. & Dalziel (*Leguminosae*)	[208]	West Africa
(6)	Ursolic acid	Terpenoids	Leaves of *Curtisia dentata* (Burm.f.) C.A.Sm (*Cornaceae*)	[209]	Southern Africa
(7)	Lupeol				
(8)	Dichapetalin A	Terpenoids	Roots of *Dichapetalum filicaule* Breteler (*Dichapetalaceae*)	[210]	West Africa
(9)	Dichapetalin X				
(10)	Glycerol monostearate				
(11)	Thymol	Monoterpenoid	Essential oils of *Thymus vulgaris* L. (*Lamiaceae*)	[211]	West Tropical Africa
(12)	Andrographolide	Terpenoid	Leaves of *Andrographis paniculata* (Burm.f.) Nees (*Acanthaceae*)	[212]	East and South Africa
(13)	Totarol	Terpenoid	Berries of *Juniperus procera* Hochst. Endl. (*Cupressaceae*)	[213]	East Africa
(14)	(3R, 6R)-Linalool oxide acetate	Monoterpenoid			
(15)	Borneol	Monoterpenoid	Aerial parts of *Zanthoxylum simulans* Hance (*Rutaceae*)	[214]	West, Central and East Africa
(16)	*β*-Elemene				
(17)	8(14),15-Sandaracopimaradiene-7*α*,18-diol	Diterpene	Leaves of *Tetradenia riparia* (Hochst.) Codd (*Lamiacea*e)	[215]	East and South Africa
(18)	Terpinen-4-ol (4-carvomenthenol)	Monoterpenoid	Essential oils *of Melaleuca alternifolia* (Maiden & Betche) Cheel (*Myrtaceae*)	[216]	South Africa
(19)	Warburganal	Sesquiterpene	Leaves of *Warburgia ugandensis* Sprague (*Canellaceae*)	[217]	East and South Africa
(20)	Polygodial				
(21)	2-Decanone	t is ok mistake from me	Essential oils from the aerial parts of *Ruta chalepensis* L. (*Meliaceae*)	[218]	North Africa
(22)	2-Nonanone				
(23)	2-Undecanone				
(24)	(E,E)-2-4-Decadienal				
(25)	Furfural				
(26)	Salicylaldehyde				
(27)	Eryngial	Carbonal	Whole herb of *Eryngium foetidum* Walter (*Apiaceae*)	[219]	Africa
(28)	tr-Cinnamaldehyde	Carbonal	Bark of *Cinnamomum verum* J. Presl. (*Lauraceae*)	[220]	Tropical Africa
(29)	Acetogenin	Polyketide	Seeds of *Annona squamosa* Delile (*Annonaceae*)	[221]	West Tropical Africa
(30)	(+)-Epicatechin-3-O-gallate	Proanthocyanidins	Fruits of *Acacia nilotica* (L.) Delile (*Fabaceae*)	[110]	Tropical Africa
(31)	(+)-Catechin-3-O-gallate				
(32)	(+)-Gallocatechin				
(33)	(−)-Epigallocatechin				
(34)	(−)-Epigallocatechin-3-O-gallate				
(35)	Prodelphinidins	Proanthocyanidins	Flowers of *Trifolium repens* L. (*Papilionaceae*)	[203]	South Africa
(36)	Ellagic acid	Simple phenolics	Leaves and stem bark of *Anogeissus leiocarpus* (DC.) Guill. & Perr. (*Combretaceae*)	[15]	Tropical, Central and East Africa
(37)	Gentisic acid				
(38)	Gallic acid	Simple phenolic	Fruits of *Caesalpinia coriaria* (Jacq.) Willd. (*Fabaceae*)	[222]	West Africa
(39)	Caffeic acid	Cinnamic acid-like derivatives (phenolics)	Leaves of *Acacia cochliacantha* Willd. (*Fabaceae*)	[223]	Africa
(40)	Ferulic acid				
(41)	p-Coumaric acid		Leaves of *Senegalia gaumeri* (S.F. Blake) Britton & Rose. (*Fabaceae*)	[224]	North-West Africa
(42)	6-Gingerol	Phenolics	Rhizomes of *Zingiber officinale* Roscoe (*Zingiberaceae*)	[225]	Tropical Africa
(43)	6-Shogaol				
(44)	10-Gingerol				
(45)	10-Shogaol				
(46)	Hexahydrocurcumin				
(47)	Chlorogenic acid	Phenolic	Aerial parts of *Tagetes filifolia* Lag. (*Compositae*)	[226]	Southern Africa
(48)	Joazeiroside B	Saponins	Stem bark of *Ziziphus joazeiro* Mart. (*Rhamnaceae*)	[210]	North Africa
(49)	Lotoside A				
(50)	Urs-19(29)- en-3-yl acetate	Saponins	Latex from the stem and leaves of *Calotropis procera* (Aiton) Dryand. (*Apocynaceae*)	[227]	North and Tropical Africa
(51)	(3*β*)-Urs-19(29)-en-3-ol				
(52)	1-(2′,5′-dimethoxyphenyl)-glycerol				
(53)	Tribulosin	Spirostanol saponins	Whole plants of *Tribulus terrestris* L. (*Zygophyllaceae*)	[228]	Tropical Africa
(54)	*β*-Sitosterol-D-glucoside				
(55)	Dioscin	Steroidal saponins	Rhizomes of *Paris polyphylla* Sm. (*Melanthiaceae*)	[229]	South Africa
(56)	Polyphyllin D				
(57)	Avenacoside	Steroidal saponin	Seeds of *Avena sativa* L. (*Poaceae*)	[230]	Tropical Africa
(58)	Uzarigenin	Cardenolides	Branches of *Nerium indicum* Mill. (*Apocynaceae*)	[231]	North Africa
(59)	Cardenolide N-1				
(60)	3*β*-O-(*β*-D-Diginosyl)-14,15*α*-dihydroxy-5*α*-card-20(22)-enolide				
(61)	Chelerythrine	Alkaloid	Aerial parts of *Chelidonium majus* L. (*Papaveraceae*)	[232]	Northern Africa
(62)	Piperine	Alkaloid	Stems of *Piper sylvaticum* Roxb. (*Piperaceae*)	[233]	West Africa
(63)	6-Methoxydihydro-sanguinarine	Alkaloids	Aerial parts of *Macleaya macrocarpa* (x kewensis) Turill (*Papaveraceae*)	[234]	North and South Africa
(64)	Sanguinarine				
(65)	(S)-Dicentrine	Aporphine alkaloids	Aerial parts of *Cissampelos capensis* L. f. (*Menispermaceae*)	[235]	Southern Africa
(66)	(S)-Neolitsine				
(67)	Melicopicine	Acridine alkaloids	Root bark of *Teclea trichocarpa* Engl. (*Rutaceae*)	[93]	East Africa
(68)	6-Methoxytecleanthine				
(69)	Deguelin	Isoflavonoids	Stem bark of *Mundulea sericea* (Wild.) A. Chev. (*Leguminosae*)	[216]	Southern Africa
(70)	Rutin	Flavonoids	Aerial hay of *Onobrychis viciifolia* Scop. (*Fabaceae*)	[236]	Tropical Africa
(71)	Nicotiflorin				
(72)	Narcissin				
(73)	Luteolin	Flavonoid	Aerial parts of *Ajania nubigena* (Wall.) C. Shih (*Compositae*)	[237]	Africa
(74)	Galangal acetate	Flavonoid	Seeds of *Torreya grandis* (*Semen torreyae*) Fortune ex Lindl. (*Taxaceae*)	[33]	Tropical West Africa
(75)	Miogadial				
(76)	Epicatechin	Flavan-3-ol	Seeds of *Persea americana* Mill. (*Lauraceae*)	[238]	West Africa
(77)	(−)-Epigallocatechin-(2*β*⟶O⟶7′,4*β*⟶8′)-epicatechin-3′-O-gallate	Dimeric flavan-3-ol	Leaves of *Camellia sinensis* (L.) Kuntze (*Theaceae*)	[239]	East Africa
(78)	Benzyl isothiocyanate	Isothiocynates	Seeds of *Carica papaya L*. (*Caricaceae*)	[240]	West, Central and East Africa
(79)	Bruceine A	Quassinoid	Dried fruits of *Brucea javanica* (L.) Merr. (*Simaroubaceae*)	[113]	East Africa
(80)	Bruceine D				
(81)	Farnesol	Prenyl alcohols	Aerial parts of *Matricaria chamomilla* Blanco (*Compositae*)	[241]	Northern and Southern Africa
(82)	Nerolidol				
(83)	2H-Chromen-2-one	Coumarin	Leaves of *Gliricidia sepium* (Jacq.) Walp. (*Fabaceae*)	[242]	West Tropical Africa
(84)	Mimosine	Amino acid	Leaves of *Leucaena leucocephala* (Lam.) de Wit. (*Fabaceae*)	[243]	Southern Africa
(85)	*α*-Linolenic acid	Fatty acid	Leaves of *Warburgia ugandensis* Sprague (*Canellaceae*)	[217]	East and South Africa

## Data Availability

No data were used to support this study.

## Abbreviations

DCM: Dichloromethane
EC_50_: Half maximal effective concentration
ED_50_: Half maximal effective dose
EHIA: Egg hatch inhibition assay
EPG: Egg per gram
FEC: Faecal egg count
FECR: Faecal egg count reduction
GIN: Gastrointestinal nematodes
IC_50_: Half maximal inhibitory concentration
LC_50_: Half maximal lethal concentration
LD_50_: Half maximal lethal dose
LMIA: Larval migration inhibition assay
MDA: Mass drug administration
QSAR: Quantitative structure-activity relations studies
STH: Soil-transmitted helminthiases
WHO: World Health Organization.

## References

[1] Bharti B., Bharti S., Khurana S. (2018). Worm infestation: diagnosis, treatment and prevention. *Indian Journal of Pediatrics*.

[2] Who (2020). *Ending the Neglect to Attain the Sustainable Development Goals: A Road Map for Neglected Tropical Diseases 2021–2030*.

[3] WHO (2020). *Neglected Tropical Diseases − Summary*.

[4] Lustigman S., Prichard R. K., Gazzinelli A. (2012). A research agenda for helminth diseases of humans: the problem of helminthiases. *PLoS Neglected Tropical Diseases*.

[5] Liu M., Panda S. K., Luyten W. (2020). Plant-based natural products for the discovery and development of novel anthelmintics against nematodes. *Biomolecules*.

[6] Goku P. E., Orman E., Quartey A. N. K., Ansong G. T., Asare-Gyan E. B. (2020). Comparative evaluation of the in vitro anthelminthic effects of the leaves, stem, and seeds of carica papaya (linn) using the Pheretima posthuma model. *Evidence-based Complementary and Alternative Medicine*.

[7] Garcia-Bustos J. F., Sleebs B. E., Gasser R. B. (2019). An appraisal of natural products active against parasitic nematodes of animals. *Parasites & Vectors*.

[8] Romero-Benavides J. C., Ruano A. L., Silva-Rivas R., Castillo-Veintimilla P., Vivanco-Jaramillo S., Bailon-Moscoso N. (2017). Medicinal plants used as anthelmintics: ethnomedical, pharmacological, and phytochemical studies. *European Journal of Medicinal Chemistry*.

[9] Vercruysse J., Albonico M., Behnke J. M. (2011). Is anthelmintic resistance a concern for the control of human soil-transmitted helminths?. *International Journal for Parasitology: Drugs and Drug Resistance*.

[10] Who (2017). *Integrating Neglected Tropical Diseases into Global Health and Development: Fourth WHO Report on Neglected Tropical Diseases*.

[11] Manke M. B., Dhawale S. C., Jamkhande P. G. (2015). Helminthiasis and medicinal plants: a review. *Asian Pacific Journal of Tropical Disease*.

[12] Stuyver L. J., Levecke B. (2021). The role of diagnostic technologies to measure progress toward who 2030 targets for soil-transmitted helminth control programs. *PLoS Neglected Tropical Diseases*.

[13] Hotez P. J., Brindley P. J., Bethony J. M., King C. H., Pearce E. J., Jacobson J. (2008). Helminth infections: the great neglected tropical diseases. *The Journal of Clinical Investigation*.

[14] WHO (2020). *WHO Factsheet: Soil-Transmitted Helminthiases*.

[15] Ndjonka D., Abladam E. D., Djafsia B., Ajonina-Ekoti I., Achukwi M. D., Liebau E. (2013). Anthelmintic activity of phenolic acids from the axlewood tree Anogeissus leiocarpus on the filarial nematode Onchocerca ochengi and drug-resistant strains of the free-living nematode *Caenorhabditis elegans*. *Journal of Helminthology*.

[16] Preston S., Korhonen P. K., Mouchiroud L. (2017). Deguelin exerts potent nematocidal activity via the mitochondrial respiratory chain. *The FASEB Journal*.

[17] Reyes-Guerrero D. E., Olmedo-Juárez A., Mendoza-De Gives P. (2021). Control y prevención de nematodosis en pequeños rumiantes: antecedentes, retos y perspectivas en México. *Revista Mexicana De Ciencias Pecuarias*.

[18] Joshi B., Panda S. K., Jouneghani R. S. (2020). Antibacterial, antifungal, antiviral, and anthelmintic activities of medicinal plants of Nepal selected based on ethnobotanical evidence. *Evidence-based Complementary and Alternative Medicine*.

[19] Humphries D., Nguyen S., Boakye D., Wilson M., Cappello M. (2012). The promise and pitfalls of mass drug administration to control intestinal helminth infections. *Current Opinion in Infectious Diseases*.

[20] Olsen A., Lieshout L. V., Marti H. (2009). Strongyloidiasis — the most neglected of the neglected tropical diseases ?. *Transactions of the Royal Society of Tropical Medicine & Hygiene*.

[21] Geary T. G. (2012). Are new anthelmintics needed to eliminate human helminthiases?. *Current Opinion in Infectious Diseases*.

[22] Mali R. G., Mehta A. A. (2008). A review on anthelmintic plants. *Indian Journal of Natural Products and Resources*.

[23] Tariq K. A., Tantry M. A. (2012). Preliminary studies on plants with anthelmintic properties in kashmir the north-west temperate himalayan region of India. *Chinese Medicine*.

[24] Williams A. R., Soelberg J., Jäger A. K. (2016). Anthelmintic properties of traditional African and Caribbean medicinal plants: identification of extracts with potent activity against *Ascaris suum* in vitro. *Parasite*.

[25] Agyare C., Asase A., Lechtenberg M., Niehues M., Deters A., Hensel A. (2009). An ethnopharmacological survey and in vitro confirmation of ethnopharmacological use of medicinal plants used for wound healing in Bosomtwi-Atwima-Kwanwoma area, Ghana. *Journal of Ethnopharmacology*.

[26] Tabi M. M., Powell M., Hodnicki D. (2006). Use of traditional healers and modern medicine in Ghana. *International Nursing Review*.

[27] Who (2019). *WHO Global Report on Traditional and Complementary Medicine 2019*.

[28] Rajeswari V. D. (2014). Anthelmintic activity of plants: a review. *Research Journal of Phytochemistry*.

[29] Fabricant D. S., Farnsworth N. R. (2001). The value of plants used in traditional medicine for drug discovery. *Environmental Health Perspectives*.

[30] Waterman C., Smith R. A., Pontiggia L., Dermarderosian A. (2010). Anthelmintic screening of Sub-Saharan African plants used in traditional medicine. *Journal of Ethnopharmacology*.

[31] Newman D. J., Cragg G. M. (2020). Natural products as sources of new drugs over the nearly four decades from 01/1981 to 09/2019. *Journal of Natural Products*.

[32] Waller P. J., Baker R. L., Githiori J. B., Ho J. (2004). Evaluation of anthelmintic properties of some plants used as livestock dewormers against *Haemonchus contortus* infections in sheep. *Parasitology*.

[33] Liu M., Kipanga P., Mai A. H. (2018). Bioassay-guided isolation of three anthelmintic compounds from Warburgia ugandensis Sprague subspecies ugandensis, and the mechanism of action of polygodial. *International Journal for Parasitology*.

[34] Geerts S., Gryseels B. (2001). Anthelmintic resistance in human helminths: a review. *Tropical Medicine and International Health*.

[35] Peregrine A. S., Molento M. B., Kaplan R. M., Nielsen M. K. (2014). Anthelmintic resistance in important parasites of horses: does it really matter?. *Veterinary Parasitology*.

[36] Traversa D., Samson-himmelstjerna G. V., Demeler J. (2009). Parasites & Vectors Anthelmintic resistance in cyathostomin populations from horse yards in Italy, United Kingdom and Germany. *Parasites & Vectors*.

[37] Jimenez Castro P. D., Howell S. B., Schaefer J. J., Avramenko R. W., Gilleard J. S., Kaplan R. M. (2019). Multiple drug resistance in the canine hookworm Ancylostoma caninum: an emerging threat?. *Parasites & Vectors*.

[38] Raza A., Qamar A. G., Hayat K., Ashraf S., Williams A. R. (2019). Anthelmintic resistance and novel control options in equine gastrointestinal nematodes. *Parasitology*.

[39] Van Wyk J. A. (2001). Refugia - overlooked as perhaps the most potent factor concerning the development of anthelmintic resistance. *Onderstepoort Journal of Veterinary Research*.

[40] Geary T. G., Thompson D. P. (2001). *Caenorhabditis elegans*: how good a model for veterinary parasites?. *Veterinary Parasitology*.

[41] Gilleard J. S. (2013). *Haemonchus contortus* as a paradigm and model to study anthelmintic drug resistance. *Parasitology*.

[42] Kotze A. C., Hunt P. W., Skuce P. (2014). Recent advances in candidate-gene and whole-genome approaches to the discovery of anthelmintic resistance markers and the description of drug/receptor interactions. *International Journal for Parasitology: Drugs and Drug Resistance*.

[43] Stuchlíková L. R., Matoušková P., Vokřál I. (2018). Metabolism of albendazole, ricobendazole and flubendazole in *Haemonchus contortus* adults: sex differences, resistance-related differences and the identification of new metabolites. *International Journal for Parasitology: Drugs and Drug Resistance*.

[44] Kwa M. S. G., Kooyman F. N. J., Boersema J. H., Roos M. H. (1993). Effect of selection for benzimidazole resistance in *Haemonchus contortus* on *β*-tubulin isotype 1 and isotype 2 genes. *Biochemical and Biophysical Research Communications*.

[45] Kwa M. S. G., Veenstra J. G., Roos M. H. (1994). Benzimidazole resistance in *Haemonchus contortus* is correlated with a conserved mutation at amino acid 200 in *β*-tubulin isotype 1. *Molecular and Biochemical Parasitology*.

[46] Kwa M. S. G. G., Veenstra J. G., Van Dijk M., Roos M. H., Roos M. H. (1995). *β*-Tubulin genes from the parasitic nematode *Haemonchus contortus* modulate drug resistance in *Caenorhabditis elegans*. *Journal of Molecular Biology*.

[47] Williams A. R., Fryganas C., Ramsay A., Mueller-Harvey I., Thamsborg S. M. (2014). Direct anthelmintic effects of condensed tannins from diverse plant sources against *Ascaris suum*. *PLoS One*.

[48] Zirintunda G., Biryomumaisho S., Kasozi K. I. (2021). Emerging anthelmintic resistance in poultry: can ethnopharmacological approaches offer a solution?. *Frontiers in Pharmacology*.

[49] Githiori J. B., Athanasiadou S., Thamsborg S. M. (2006). Use of plants in novel approaches for control of gastrointestinal helminths in livestock with emphasis on small ruminants. *Veterinary Parasitology*.

[50] Tinkler S. H. (2020). Preventive chemotherapy and anthelmintic resistance of soil-transmitted helminths – can we learn nothing from veterinary medicine?. *One Health*.

[51] Vuuren S. F. (2018). A review of the traditional use of southern African medicinal plants for the treatment of selected parasite infections affecting humans. *Journal of Ethnopharmacology*.

[52] Enejoh S. O., Suleiman M. M. (2017). Anthelmintics and their application in veterinary medicine. *Research in Medical & Engineering Sciences*.

[53] Smout M. J., Kotze A. C., Mccarthy J. S., Loukas A. (2010). A novel high throughput assay for anthelmintic drug screening and resistance diagnosis by real-time monitoring of parasite motility. *PLoS Neglected Tropical Diseases*.

[54] Wink M. (2012). Medicinal plants: a source of anti-parasitic secondary metabolites. *Molecules*.

[55] Hilou A., Rappez F., Duez P. (2015). Ethnoveterinary management of cattle helminthiasis among the Fulani and the Mossi (Central Burkina Faso): plants used and modes of use. *International Journal of Brain and Cognitive Sciences*.

[56] C Campbell W. (2012). History of avermectin and ivermectin, with notes on the history of other macrocyclic lactone antiparasitic agents. *Current Pharmaceutical Biotechnology*.

[57] Hu Y., Xiao S. H., Aroian R. V. (2009). The new anthelmintic tribendimidine is an L-type (Levamisole and Pyrantel) nicotinic acetylcholine receptor agonist. *PLoS Neglected Tropical Diseases*.

[58] Onakpoya I. J. (2019). Antihelminthic drugs. *Side Effects of Drugs Annual*.

[59] Harvey A. L., Edrada-Ebel R., Quinn R. J. (2015). The re-emergence of natural products for drug discovery in the genomics era. *Nature Reviews Drug Discovery*.

[60] Behnke J. M., Buttle D. J., Stepek G., Lowe A., Duce I. R. (2008). Developing novel anthelmintics from plant cysteine proteinases. *Parasites & Vectors*.

[61] Baell J. B. (2016). Feeling nature’s PAINS: natural products, natural product drugs, and Pan assay interference compounds (PAINS). *Journal of Natural Products*.

[62] Baell J. B., Nissink J. W. M. (2018). Seven year itch: pan-assay interference compounds (PAINS) in 2017 - utility and limitations. *ACS Chemical Biology*.

[63] Boukandou Mounanga M., Mewono L., Aboughe Angone S. (2015). Toxicity studies of medicinal plants used in sub-Saharan Africa. *Journal of Ethnopharmacology*.

[64] Levine M., Ruha A. M., Graeme K., Brooks D. E., Canning J., Curry S. C. (2011). Toxicology in the ICU - Part 3: natural toxins. *Chest*.

[65] Ali N., Shah S. W. A., Shah I., Ahmed G., Ghias M., Khan I. (2011). Cytotoxic and anthelmintic potential of crude saponins isolated from Achillea Wilhelmsii C. Koch and Teucrium Stocksianum boiss. *BMC Complementary and Alternative Medicine*.

[66] Okem A., Finnie J. F., Van Staden J. (2012). Pharmacological, genotoxic and phytochemical properties of selected South African medicinal plants used in treating stomach-related ailments. *Journal of Ethnopharmacology*.

[67] Newman D. J., Cragg G. M. (2016). Natural products as sources of new drugs from 1981 to 2014. *Journal of Natural Products*.

[68] Kalmobé J., Ndjonka D., Boursou D., Vildina J. D., Liebau E. (2017). Phytochemical analysis and in vitro anthelmintic activity of Lophira lanceolata (Ochnaceae) on the bovine parasite Onchocerca ochengi and on drug resistant strains of the free-living nematode *Caenorhabditis elegans*. *BMC Complementary and Alternative Medicine*.

[69] Aremu A. O., Fawole O. A., Chukwujekwu J. C., Light M. E., Finnie J. F., Van Staden J. (2010). In vitro antimicrobial, anthelmintic and cyclooxygenase-inhibitory activities and phytochemical analysis of Leucosidea sericea. *Journal of Ethnopharmacology*.

[70] Karonen M., Ahern J. R., Legroux L. (2020). Ellagitannins inhibit the exsheathment of *Haemonchus contortus* and Trichostrongylus colubriformis larvae: the efficiency increases together with the molecular size. *Journal of Agricultural and Food Chemistry*.

[71] Karole S., Shrivastava S., Thomas S. (2019). Polyherbal formulation concept for synergic action: a review. *Journal of Drug Delivery and Therapeutics*.

[72] Gomez-Galera S., Pelacho A. M., Gene A., Capell T., Christou P. (2007). The genetic manipulation of medicinal and aromatic plants. *Plant Cell Reports*.

[73] Cos P., Vlietinck A. J., Berghe D. V., Maes L. (2006). Anti-infective potential of natural products: how to develop a stronger in vitro “proof-of-concept. *Journal of Ethnopharmacology*.

[74] Oliveira Santos F., Ponce Morais Cerqueira A., Branco A., José Moreira Batatinha M., Borges Botura M. (2019). Anthelmintic activity of plants against gastrointestinal nematodes of goats: a review. *Parasitology*.

[75] Aremu A. O., Finnie J. F., Van Staden J. (2012). Potential of South African medicinal plants used as anthelmintics – their ef fi cacy, safety concerns and reappraisal of current screening methods. *South African Journal of Botany*.

[76] Simpkin K. G., Coles G. C. (2007). The use of *Caenorhabditis elegans* for anthelmintic screening. *Journal of Chemical Technology and Biotechnology*.

[77] Nouri F., Nourollahi-Fard S. R., Foroodi H. R., Sharifi H. (2014). In vitro anthelmintic effect of Tobacco (Nicotiana tabacum) extract on parasitic nematode, Marshallagia marshalli. *Journal of Parasitic Diseases*.

[78] Verkman A. S. (2004). Drug discovery in academia. *American Journal of Physiology - Cell Physiology*.

[79] Spiegler V., Liebau E., Hensel A. (2017). Medicinal plant extracts and plant-derived polyphenols with anthelmintic activity against intestinal nematodes. *Natural Product Reports*.

[80] Yadav B., Wennerberg K., Aittokallio T., Tang J. (2015). Searching for drug synergy in complex dose-response landscapes using an interaction potency model. *Computational and Structural Biotechnology Journal*.

[81] Eguale T., Giday M. (2009). In vitro anthelmintic activity of three medicinal plants against *Haemonchus contortus*. *International Journal of Green Pharmacy*.

[82] Katiki L. M., Ferreira J. F. S., Zajac A. M. (2011). *Caenorhabditis elegans* as a model to screen plant extracts and compounds as natural anthelmintics for veterinary use. *Veterinary Parasitology*.

[83] Adamu M., Naidoo V., Eloff J. N. (2013). Efficacy and toxicity of thirteen plant leaf acetone extracts used in ethnoveterinary medicine in South Africa on egg hatching and larval development of *Haemonchus contortus*. *BMC Veterinary Research*.

[84] Githiori J. B., Höglund J., Waller P. J., Leyden Baker R. (2003). Evaluation of anthelmintic properties of extracts from some plants used as livestock dewormers by pastoralist and smallholder farmers in Kenya against Heligmosomoides polygyrus infections in mice. *Veterinary Parasitology*.

[85] Kumarasingha R., Karpe A. V., Preston S. (2016). Metabolic profiling and in vitro assessment of anthelmintic fractions of Picria fel-terrae Lour. *International Journal for Parasitology: Drugs and Drug Resistance*.

[86] Thoithi G. N., Maingi N., Karume D., Gathumbi P. K., Mwangi J. W., Kobwage I. O. (2002). Anthelminitic and other Pharmacological activities of the root bark extracts of Albizia anthelmintica Brongn. *East and Central African Journal of Pharmaceutical Sciences*.

[87] Nwosu C. O., Onu E., Alalade E. (2008). Anthelmintic efficacy of aqueous extract of seeds of some plants used traditionally as spices in Nigeria. *Global Journal of Medical Sciences*.

[88] Simon K., Jegede O. (2010). Anthelmintic properties of Afzelia africana&lt sw: an in-vitro egg hatch assay. *Animal Production Research Advances*.

[89] Adama K., Gaston B. A. M., Hamidou H. ., T., Amadou T., Laya S. (2009). In vitro anthelmintic effect of two medicinal plants (Anogeissus leiocarpus and Daniellia oliveri) on *Haemonchus contortus*, an abosomal nematode of sheep in Burkina Faso. *African Journal of Biotechnology*.

[90] Adamu M., Oshadu O. D., Ogbaje C. I. (2010). Anthelminthic efficacy of aqueous extract of Acanthus montanus leaf against strongylid nematodes of small ruminants. *African Journal of Traditional, Complementary and Alternative Medicines: AJTCAM*.

[91] Onyenwe I. W., Ngongeh L. A., Udekwu C. C., Ezeugwu G. O. (2010). Preliminary studies on the anthelmintic effects of ethanolic extract of Garcinia kola (Heckel) seed and methanolic extract of sacoglottis gabonensis (baillon) stem bark on Heligmosomoides bakeri larvae in nsukka, Nigeria. *Global Journal of Pure and Applied Sciences*.

[92] Ademola I. O., Eloff J. N. (2011). In vitro anthelmintic effect of Anogeissus leiocarpus (DC.) Guill. & Perr. leaf extracts and fractions on developmental stages of *Haemonchus contortus*. *African Journal of Traditional, Complementary and Alternative Medicines: AJTCAM*.

[93] Muema S. M., Abuga K. O., Yenesew A., Thoithi G. N. (2014). Phytochemical and anthelmintic study of the root bark of teclea trichocarpa, engl. (Rutaceae). *East and Central African Journal of Pharmaceutical Sciences*.

[94] Odhong C., Wahome R. G., Vaarst M. (2014). In vitro anthelmintic effects of crude aqueous extracts of Tephrosia vogelii, Tephrosia villosa and Carica papaya leaves and seeds. *African Journal of Biotechnology*.

[95] Ademola I. O., Fagbemi B. O., Idowu S. O. (2008). Anthelmintic efficacy of Nauclea latifolia&lt extract against gastrointestinal nematodes of sheep:in vitro&lt and in vivo&lt studies. *African Journal of Traditional, Complementary and Alternative Medicines*.

[96] Pone J., Bilong B. C. F., Mpoame M., Ngwa C. F., Coles G. C. (2006). In vitro activity of ethanol, cold water and hot water extracxts of the bark of Canthium mannii (Rubiaceae) stem on agasints Ancylostoma caninum eggs. *East and Central African Journal of Pharmaceutical Sciences*.

[97] Bachaya H. A., Iqbal Z., Khan M. N., Sindhu Z. ud D., Jabbar A. (2009). Anthelmintic activity of Ziziphus nummularia (bark) and Acacia nilotica (fruit) against Trichostrongylid nematodes of sheep. *Journal of Ethnopharmacology*.

[98] Ilango K., Xavier R. A., Subburaju T. (2011). Original research article open access anthelmintic activity of extracts of aerial parts of Tephrosia spinosa (L.f.) pres. *International Journal of Health Research*.

[99] Danquah C. A., Koffuor G. A., Annan K., Ketor E. C. (2012). The anthelmintic activity of Vernonia amygdalina and Alstonia boonei De Wild. *Journal of Medicine and Biomedical Sciences*.

[100] Adu F., Agyare C., Sam G. H., Duah Boakye Y., Boamah V. E. (2018). Anthelmintic resistance modifying properties of extracts of Cyperus difformis L. (Cyperiaceae). *Investigational Medicinal Chemistry and Pharmacology*.

[101] Asante-Kwatia E., Mensah A. Y., Forkuo A. D. (2021). The Ghanaian flora as a potential source of anthelmintic and anti-schistosomal agents. *Natural Medicinal Plants Among*.

[102] Thorn G. W., Adams R. D., Braunwald K., Isselbacher K. J., Petersdorf R. G. (1997). Harrison’s. Principles of Internal Medicine. In McGraw Hill Co.

[103] Vigar Z. (1984). Atlas of Medicinal Parasitology. *In P. G. Publication House*.

[104] Chittoor S. M., Binny A. J. R., Yadlapalli S. K., Cheruku A., Dandu C., Nimmanapalli Y. (2012). Anthelmintic and antimicrobial studies of Drimia indica (Roxb.) Jessop. bulb aqueous extracts. *Journal of Pharmacy Research*.

[105] Karumi E. W., Maitai1 C. K., Okalebo F. A. (2013). Anthelmintic and antibacterial activity of hagenia abyssinica (bruce) J.F. Gmel (rosaceae). *East and Central African Journal of Pharmaceutical Sciences*.

[106] Gagman A. H., Ahmad H., Ahmad N., Izzaudin I., Him N. (2019). The efficacy of Vitex doniana and Boswellia dalzielii against egg hatch of *Caenorhabditis elegans*. *Bayero Journal of Pure and Applied Sciences*.

[107] Kumarasingha R., Palombo E. A., Bhave M. (2014). Enhancing a search for traditional medicinal plants with anthelmintic action by using wild type and stress reporter *Caenorhabditis elegans* strains as screening tools. *International Journal for Parasitology*.

[108] Asha M. K., Prashanth D., Murali B., Padmaja R., Amit A. (2001). Anthelmintic activity of essential oil of Ocimum sanctum and eugenol. *Fitoterapia*.

[109] Prashanth D., Asha M. K., Amit A., Padmaja R. (2001). Anthelmintic activity of Butea monosperma. *Fitoterapia*.

[110] Dikti Vildina J., Kalmobe J., Djafsia B., Schmidt T. J., Liebau E., Ndjonka D. (2017). Anti-Onchocerca and anti-Caenorhabditis activity of a hydro-alcoholic extract from the fruits of Acacia nilotica and some proanthocyanidin derivatives. *Molecules*.

[111] Ameen S. A., Azeez O. M., Baba Y. A. (2018). Anthelmintic potency of carica papaya seeds against gastro-intestinal helminths in red sokoto goat. *Ceylon Journal of Science*.

[112] Max R. A., Kimambo A. E., Kassuku A. A. A., Mtenga L. A. La, Buttery P. J. (2007). Effect of tanniniferous browse meal on nematode faecal egg counts and internal parasite burdens in sheep and goats. *South African Journal of Animal Science*.

[113] Wang Y., Wu Z. F., Wang G. X. (2011). In vivo anthelmintic activity of bruceine A and bruceine D from Brucea javanica against Dactylogyrus intermedius (Monogenea) in goldfish (*Carassius auratus*). *Veterinary Parasitology*.

[114] Hosseinzadeh S., Ghalesefidi M. J., Azami M., Mohaghegh M. A., Hejazi S. H., Ghomashlooyan M. (2016). In vitro and in vivo anthelmintic activity of seed extract of Coriandrum sativum compared to Niclosamid against Hymenolepis nana infection. *Journal of Parasitic Diseases*.

[115] Niaz S., Akhtar T., Shams S. (2015). Treatment of bovine schistosoiasis with medicinal plant Vernonia anthelmintica (Kaliziri), an alternative approach. *African Journal of Traditional, Complementary and Alternative Medicines*.

[116] Ameen S. A. (2011). Anthelmintic efficacy of pawpaw (Carica papaya) seeds in commercial layers. *African Journal of Biotechnology*.

[117] Nweze N. E., Asuzu I. U. (2006). The anthelmintic effects of Buchholzia coriacea seed. *Nigerian Veterinary Journal*.

[118] Nzeakor T. A., Udobi M. I., Eke I. G. (2021). Evidence-based investigations into the ethnoveterinary use of Mimosa pudica L. (Fabaceae) as an anthlemintic. *Tropical Journal of Pharmaceutical Research*.

[119] Botura M. B., Silva G. D., Lima H. G. (2011). In vivo anthelmintic activity of an aqueous extract from sisal waste (Agave sisalana Perr.) against gastrointestinal nematodes in goats. *Veterinary Parasitology*.

[120] Githiori J. B., Höglund J., Waller P. J., Baker R. L. (2002). Anthelmintic activity of preparations derived from Myrsine africana and Rapanea melanophloeos against the nematode parasite, *Haemonchus contortus*, of sheep. *Journal of Ethnopharmacology*.

[121] Iqbal Z., Lateef M., Jabbar A., Muhammad G., Khan M. N. (2005). Anthelmintic activity of Calotropis procera (Ait.) Ait. F. flowers in sheep. *Journal of Ethnopharmacology*.

[122] Ademola I. O., Eloff J. N. (2011). Anthelmintic efficacy of cashew (Anarcadium occidentale L.) on in vitro susceptibility of the ova and larvae of *Haemonchus contortus*. *African Journal of Biotechnology*.

[123] Rosa S. S., Santos F. O., Lima H. G. (2018). In vitro anthelmintic and cytotoxic activities of extracts of Persea willdenovii Kosterm (Lauraceae). *Journal of Helminthology*.

[124] Fouche G., Sakong B. M., Adenubi O. T. (2016). Anthelmintic activity of acetone extracts from South African plants used on egg hatching of *Haemonchus contortus*. *Onderstepoort Journal of Veterinary Research*.

[125] Assiak I. E., Olufemi B. E., Ayonde G. O., Onigemo M. (2002). Preliminary studies on the effects of Amaranthus spinosus Leaf Extract as an Anthelmintic in growing pigs. *Tropical Veterinarian*.

[126] Sujon M. A., Mostofa M., Jahan M. S., Das A. R., Rob S. (1970). Studies on medicinal plants against gastroinstestinal nematodes of goats. *Bangladesh Journal of Veterinary Medicine*.

[127] Jabbar A., Zaman M. A., Iqbal Z., Yaseen M., Shamim A. (2007). Anthelmintic activity of *Chenopodium album* (L.) and Caesalpinia crista (L.) against trichostrongylid nematodes of sheep. *Journal of Ethnopharmacology*.

[128] Gbolade A. A., Adeyemi A. A. (2008). Anthelmintic activities of three medicinal plants from Nigeria. *Fitoterapia*.

[129] Ogedengbe A. N., Idowu S. O., Ademola I. O. (2019). Anthelmintic screening of phytomedicines using Haemonchus placei Adult motility assay. *Nigerian Journal of Pharmaceutical Research*.

[130] Ferreira L. E., Castro P. M. N., Chagas A. C. S., França S. C., Beleboni R. O. (2013). In vitro anthelmintic activity of aqueous leaf extract of Annona muricata L. (Annonaceae) against *Haemonchus contortus* from sheep. *Experimental Parasitology*.

[131] Alawa C. B. I., Adamu A. M., Gefu J. O. (2003). In vitro screening of two Nigerian medicinal plants (Vernonia amygdalina and Annona senegalensis) for anthelmintic activity. *Veterinary Parasitology*.

[132] Chisara L. V., Cosmas N. J., Chidozie H., Ikechukwu R., Chris A. N., Nnah S. (2021). Anthelmintic activities of Polyalthia longifolia leaf and stem bark extracts in Heligmosimoides bakeri infected mice. *Animal Research International*.

[133] Suleiman M. M., Mamman M., Aliu Y. O., Ajanusi J. O. (2005). Anthelmintic activity of the crude methanol extract of Xylopia aethiopica against Nippostrongylus brasiliensis in rats. *Veterinarski Arhiv*.

[134] Apenteng J. A., Ogundeyi M., Oppong E. E., Osei-asare C., Brookman-Amissah M. G. (2016). In vitro Anti-infective and Antioxidant activity of Xylopia aethiopica [Dun.] A. Rich: a comparison of the fruits and leaves extracts. *Journal of Medicinal Plants Studies*.

[135] Michael W. K., John A. A., David N. M., Bright S. A., Ivan N. D., Samuel B. A. (2016). In vitro anthelmintic activity of stem and root barks of Alstonia boonei De Wild. *Journal of Medicinal Plants Research*.

[136] Shivkar Y. M., Kumar V. L. (2003). Anthelmintic activity of latex of Calotropis procera. *Pharmaceutical Biology*.

[137] Francis A., John A. A., William G. A., George H. S., David N. M., Edna B. B. (2015). Antioxidant and in-vitro anthelminthic potentials of methanol extracts of barks and leaves of Voacanga africana and Rauwolfia vomitoria. *African Journal of Microbiology Research*.

[138] Merveille M. G. R., Célestine N. L., Brice K. A., Akhanovna M.-B. J., Yves-Alain B., Jean-Maurille O. (2021). Phytochemical study and anthelmintic activity of nine Congolese medicinal plants. *Journal of Pharmaceutical Research International*.

[139] Tariq K. A., Chishti M. Z., Ahmad F., Shawl A. S. (2009). Anthelmintic activity of extracts of Artemisia absinthium against ovine nematodes. *Veterinary Parasitology*.

[140] Iqbal Z., Lateef M., Ashraf M., Jabbar A. (2004). Anthelmintic activity of Artemisia brevifolia in sheep. *Journal of Ethnopharmacology*.

[141] Sisay A., Negesse T., Nurfeta A. (2021). Anthelminthic effects of extracts of indigenous browses from mid rift valley of Ethiopia. *Ethiopian Veterinary Journal*.

[142] Quartey A., Oppong A., Ayensu I., Apenteng J., Mintah D., Ikeani C. Synergistic in-vitro anthelmintic potentials of Vernonia amygdalina Delile stem and Carica papaya Lin. seeds.

[143] Agaie B. M., Onyeyili P. A. (2007). Anthelmintic activity of the crude aqueous leaf extracts of Anogeissus leiocarpus in sheep. *African Journal of Biotechnology*.

[144] Ndjonka D., Agyare C., Lüersen K. (2011). In vitro activity of Cameroonian and Ghanaian medicinal plants on parasitic (Onchocerca ochengi) and free-living (*Caenorhabditis elegans*) nematodes. *Journal of Helminthology*.

[145] Okpekon T., Yolou S., Gleye C. (2004). Antiparasitic activities of medicinal plants used in Ivory Coast. *Journal of Ethnopharmacology*.

[146] Jegede O. C., Abubakar M. S., George B. D. J., Ajanusi O. J., Obeta S. (2015). Anthelmintic efficacy trials using fractions of ethanolic crude extract of Anogeissus schimperi hoechst against nippostrongylus braziliensis in rats. *Nigerian Veterinary Journal*.

[147] Ademola I. O., Eloff J. N. (2010). In vitro anthelmintic activity of Combretum molle (R. Br. ex G. Don) (Combretaceae) against *Haemonchus contortus* ova and larvae. *Veterinary Parasitology*.

[148] Koné W. M., Vargas M., Keiser J. (2012). Anthelmintic activity of medicinal plants used in Côte d’Ivoire for treating parasitic diseases. *Parasitology Research*.

[149] Agyare C., Spiegler V., Sarkodie H., Asase A., Liebau E., Hensel A. (2014). An ethnopharmacological survey and in vitro confirmation of the ethnopharmacological use of medicinal plants as anthelmintic remedies in the Ashanti region , in the central part of Ghana. *Journal of Ethnopharmacology*.

[150] Spiegler V., Sendker J., Petereit F., Liebau E., Hensel A. (2015). Bioassay-Guided fractionation of a leaf extract from combretum mucronatum with anthelmintic activity: oligomeric procyanidins as the active principle. *Molecules*.

[151] Herrmann F. C., Spiegler V. (2019). *Caenorhabditis elegans* revisited by atomic force microscopy – ultra-structural changes of the cuticle, but not in the intestine after treatment with Combretum mucronatum extract. *Journal of Structural Biology*.

[152] Gagman H. A., Irwan N. A., Ahmad H. B. (2019). In vitro&lt study of the anthelmintic activity of aqueous and methanol extract of Guiera senegalensis&lt against egg hatch and larval developmental of *Caenorhabditis elegans*&lt. *Bayero Journal of Pure and Applied Sciences*.

[153] Olukotun A. B., Bello I. A., Oyewale O. A. (2018). Phytochemical and anthelmintic activity of *Terminalia catappa* (Linn) leaves. *Journal of Applied Sciences & Environmental Management*.

[154] Okpo S., Nnajekwu O. (2013). In vitro&lt anthelmintic activity of the seed extracts of three plants of the Cucurbitaceae family on Lumbricus terrestris&lt. *Journal of Pharmacy & Bioresources*.

[155] Beloin N., Gbeassor M., Akpagana K. (2005). Ethnomedicinal uses of Momordica charantia (Cucurbitaceae) in Togo and relation to its phytochemistry and biological activity. *Journal of Ethnopharmacology*.

[156] Akoto C. O., Acheampong A., Boakye Y. D., Akwata D., Okine M. (2019). In vitro anthelminthic, antimicrobial and antioxidant activities and FTIR analysis of extracts of Alchornea cordifolia leaves. *Journal of Pharmacognosy and Phytochemistry*.

[157] Gathuma J. M., Mbaria J. M., Wanyama J., Kaburia H. F. A., Mpoke L., Mwangi J. N. (2004). Efficacy of Myrsine africana, Albizia anthelmintica and Hilderbrantia sepalosa herbal remedies against mixed natural sheep helminthosis in Samburu district, Kenya. *Journal of Ethnopharmacology*.

[158] Eguale T., Tadesse D., Giday M. (2011). In vitro anthelmintic activity of crude extracts of five medicinal plants against egg-hatching and larval development of *Haemonchus contortus*. *Journal of Ethnopharmacology*.

[159] Osei Akoto C., Acheampong A., Boakye Y. D., Naazo A. A., Adomah D. H. (2020). Anti-inflammatory, antioxidant, and anthelmintic activities of Ocimum basilicum (sweet basil) fruits. *Journal of Chemistry*.

[160] Aderibigbe S. A., Idowu S. O. (2020). Anthelmintic activity of Ocimum gratissimum and Cymbopogon citratus leaf extracts against Haemonchus placei adult worm. *Journal of Pharmacy & Bioresources*.

[161] Jaradat N., Adwan L., K’aibni S., Shraim N., Zaid A. N. (2016). Chemical composition, anthelmintic, antibacterial and antioxidant effects of Thymus bovei essential oil. *BMC Complementary and Alternative Medicine*.

[162] Iqbal Z., Babar W., Sindhu Z., ud D., Abbas R. Z., Sajid M. S. (2012). Evaluation of anthelmintic activity of different fractions of Azadirachta indica A. Juss Seed Extract. *Pakistan Veterinary Journal*.

[163] Iqbal Z., Lateef M., Jabbar A., Gilani A. H. (2010). In vivo anthelmintic activity of Azadirachta indica A. Juss seeds against gastrointestinal nematodes of sheep. *Veterinary Parasitology*.

[164] Rabiu H., Subhasish M. (2011). Investigation of in vitro anthelmintic activity of Azadirachta indica Leaves. *International Journal of Drug Development & Research*.

[165] Jamra N., Das G., Singh P., Haque M. (2015). Anthelmintic efficacy of crude neem (Azadirachta indica) leaf powder against bovine strongylosis. *Journal of Parasitic Diseases*.

[166] Kausar S. (2017). In vitro evaluation of antifilarial effect of Azadirachta indica leaves extract in different solvents on the microfilariae of Setaria cervi. *Journal of Parasitic Diseases*.

[167] Nwosu C. O., Yakubu S., Ummate A., Abdullahi G. (2006). In-vitro anthelmintic efficacy of crude aqueous extracts of neem (Azadirachta indica) leaf, stem and root on nematode. *Animal Research International*.

[168] Ademola I. O., Fagbemi B. O., Idowu S. O. (2004). Evaluation of the anthelmintic activity of Khaya senegalensis extract against gastrointestinal nematodes of sheep: in vitro and in vivo studies. *Veterinary Parasitology*.

[169] Gregory L., Yoshihara E., Silva L. K. F. (2016). Anthelmintic effects of dried ground banana plant leaves (Musa spp.) fed to sheep artificially infected with *Haemonchus contortus* and Trichostrongylus colubriformis. *African Journal of Traditional, Complementary and Alternative Medicines*.

[170] Oliveira L. N., Duarte E. R., Nogueira F. A., Silva R. B. d., Faria Filho D. E. d., Geraseev L. C. (2009). Eficácia de resíduos da bananicultura sobre a inibição do desenvolvimento larval em Haemonchus spp. provenientes de ovinos. *Ciência Rural*.

[171] Nogueira F. A., Oliveira L. N., Da Silva R. B. (2012). Anthelminthic efficacy of banana crop residues on gastrointestinal nematodes of sheep: in vitro and in vivo tests. *Parasitology Research*.

[172] Marie-Magdeleine C., Boval M., Philibert L., Borde A., Archimède H. (2010). Effect of banana foliage (Musa x paradisiaca) on nutrition, parasite infection and growth of lambs. *Livestock Science*.

[173] Ezea B. O., Ogbole O. O., Ajaiyeoba E. O. (2019). In vitro&lt anthelmintic properties of root extracts of three Musa&lt species. *Journal of Pharmacy & Bioresources*.

[174] Payne V. K., Poneacute J., Kollins N. (2013). In vitro comparative effect of extracts of the seeds of Embelia rowlandii (Myrsinaceae) on the eggs and L1 and L2 larval stages of the parasitic nematode Heligmosomoides bakeri (Nematoda; Heligmosomatidae). *African Journal of Biotechnology*.

[175] Ali N., Aleem U., Ali Shah S. W. (2013). Acute toxicity, brine shrimp cytotoxicity, anthelmintic and relaxant potentials of fruits of Rubus fruticosus Agg. *BMC Complementary and Alternative Medicine*.

[176] John A. A., David N. M., Michael W. K. (2017). In vitro anti-infective and antioxidant activities of Garcinia cola Heckel and Morinda lucida Benth. *Journal of Medicinal Plants Research*.

[177] Hounzangbe-Adote S., Fouraste I., Moutairou K., Hoste H. (2005). In vitro effects of four tropical plants on the activity and development of the parasitic nematode, Trichostrongylus colubriformis. *Journal of Helminthology*.

[178] Onyeyili P. A., Nwosu C. O., Amin J. D., Jibike J. I. (2001). Anthelmintic activity of crude aqueous extract of Nauclea latifolia stem bark against ovine nematodes. *Fitoterapia*.

[179] Kiambom T., Kouam M. K., Ngangoum C. D., Kate B., Teguia A. (2020). In vivo anthelmintic effect of ginger (zingiber officinale) powder against gastointestinal nematodes of artificially infected pigs. *Archives of Veterinary Science and Medicine*.

[180] Iqbal Z., Lateef M., Akhtar M. S., Ghayur M. N., Gilani A. H. (2006). In vivo anthelmintic activity of ginger against gastrointestinal nematodes of sheep. *Journal of Ethnopharmacology*.

[181] Hussain A., Khan M. N., Iqbal Z., Sajid M. S., Khan M. K. N. (2011). Anthelmintic activity of Trianthema portulacastrum L. and Musa paradisiaca L. against gastrointestinal nematodes of sheep. *Veterinary Parasitology*.

[182] Eguale T., Tilahun G., Debella A., Feleke A., Makonnen E. (2007). In vitro and in vivo anthelmintic activity of crude extracts of Coriandrum sativum against *Haemonchus contortus*. *Journal of Ethnopharmacology*.

[183] Eguale T., Tilahun G., Debella A., Feleke A., Makonnen E. (2007). *Haemonchus contortus*: in vitro and in vivo anthelmintic activity of aqueous and hydro-alcoholic extracts of Hedera helix. *Experimental Parasitology*.

[184] Oliveira L. M. B., Bevilaqua C. M. L., Costa C. T. C. (2009). Anthelmintic activity of Cocos nucifera L. against sheep gastrointestinal nematodes. *Veterinary Parasitology*.

[185] Azando E. V. B., Hounzangbé-Adoté M. S., Olounladé P. A. (2011). Involvement of tannins and flavonoids in the in vitro effects of Newbouldia laevis and Zanthoxylum zanthoxyloïdes extracts on the exsheathment of third-stage infective larvae of gastrointestinal nematodes. *Veterinary Parasitology*.

[186] Adu O. A., Akingboye K. A., Akinfemi A. (2009). Potency of pawpaw (carica papaya) latex as an anthelmintic in poultry production. *Botany Research International*.

[187] Effendy A., Suparjo N., Ameen S., Abdullah O. (2014). Evaluation of anthhelmintic potential of pawpaw (carica papaya) seeds administered in-feed and in-water for west african dwarf (WAD) goats. *Journal of Biology, Agriculture and Healthcare*.

[188] Ameen S. A., Adedeji O. S., Ojedapo L. O., Salihu T., Fabusuyi C. O. (2010). Anthelmintic potency of pawpaw (carica papaya) seeds in West African Dwarf (WAD) sheep. *Global Veterinaria*.

[189] Attah D., Amaka J. I. (2019). In-vitro anthelmintic activity of Garcinia kola aqueous seed extract on the infective stage of strongylid nematodes of goats. *Journal of Innovative Research in Life Sciences*.

[190] Nwosu C. O., Mobee K. M., Gulani I. G., Igbokwe I. O., Ogubuaja V. O. (2006). Anthelminthic efficacy of aqueous extracts of Garcina kola&lt seed and stem bark against strongylid nematodes of small ruminants in Nigeria. *Nigerian Journal of Parasitology*.

[191] Luka J., Mbaya A. W., Biu A. A., Nwosu C. O. (2017). The effect of Diospyros mespiliformis (Ebenaceae) extracts on the haemato-biochemical parameters of Yankasa Sheep experimentally infected with *Haemonchus contortus*&lt. *Nigerian Journal of Parasitology*.

[192] Nweze N. E., Ngongeh L. A. (2007). In vitro anthelmintic activity of Anthocleista djalonensis. *Nigerian Veterinary Journal*.

[193] Kumarasingha R., Preston S., Yeo T. C. (2016). Anthelmintic activity of selected ethno-medicinal plant extracts on parasitic stages of *Haemonchus contortus*. *Parasites & Vectors*.

[194] Jegede O. C., Ajanusi J. O., Adaudi A. O., Agbede R. I. S. (2006). Anthelmintic efficacy of extracts of Spigelia anthelmia Linn on experimental Nippostrongylus braziliensis in rats. *Journal of Veterinary Science*.

[195] Asha B., Krishnappa M., Kenchappa R. (2016). In vitro anthelmintic activity of different extracts of memecylon umbellatum&ltBurm. *Science, Technology and Arts Research Journal*.

[196] Gbolade A. A., Adeyemi A. A. (2008). Investigation of in vitro anthelmintic activities of Pycnanthus angolensis and Sphenocentrum jollyanum. *Fitoterapia*.

[197] Aboagye I. F., Mensah D., Boadu F. (2015). Anthelmintic effect of moringa oleifera lam. In wild caught Achatina achatina linnaeus, 1758 from the sefwi wiawso district, Ghana. *West African Journal of Applied Ecology*.

[198] Apenteng J. A., Brookman-Amissah M. G., Osei-Asare C., Oppong E. E., Ogundeyi M. (2016a). In vitro anti-infective and antioxidant activity of plumbago zeylanica linn. *International Journal of Current Research in Biosciences and Plant Biology*.

[199] Spiegler V., Liebau E., Peppler C. (2016). A hydroalcoholic extract from paullinia pinnata LRoots exerts anthelmintic activity against free-living and parasitic nematodes. *Planta Medica*.

[200] Spiegler V. (2020). Anthelmintic A-type procyanidins and further characterization of the phenolic composition of a root extract from paullinia pinnata. *Molecules*.

[201] Ali N., Ali Shah S. W., Shah I. (2012). Anthelmintic and relaxant activities of Verbascum thapsus Mullein. *BMC Complementary and Alternative Medicine*.

[202] Iqbal Z., Lateef M., Jabbar A., Ghayur M. N., Gilani A. H. (2006). In vitro and in vivo anthelmintic activity of Nicotiana tabacum L leaves against gastrointestinal nematodes of sheep. *Phytotherapy Research*.

[203] Williams A. R., Ropiak H. M., Fryganas C., Desrues O., Mueller-Harvey I., Thamsborg S. M. (2014). Assessment of the anthelmintic activity of medicinal plant extracts and purified condensed tannins against free-living and parasitic stages of Oesophagostomum dentatum. *Parasites & Vectors*.

[204] McGaw L. J., Jäger A. K., Van Staden J., Eloff J. (2002). Isolation of *β*-asarone, an antibacterial and anthelmintic compound, from Acorus calamus in South Africa. *South African Journal of Botany*.

[205] Bosman A. A., Combrinck S., Roux-Van Der Merwe R., Botha B. M., Mccrindle R. I., Houghton P. (2004). Isolation of an anthelmintic compound from Leucosidea sericea. *South African Journal of Botany*.

[206] Fobofou S. A. T., Franke K., Sanna G. (2015). Isolation and anticancer, anthelminthic, and antiviral (HIV) activity of acylphloroglucinols, and regioselective synthesis of empetrifranzinans from Hypericum roeperianum. *Bioorganic & Medicinal Chemistry*.

[207] Carvalho V. F., Ramos L. D. A., Da Silva C. A. (2020). In vitro anthelmintic activity of Siparuna guianensis extract and essential oil against Strongyloides venezuelensis. *Journal of Helminthology*.

[208] Enwerem N. M., Okogun J. I., Wambebe C. O., Okorie D. A., Akah P. A. (2001). Anthelmintic activity of the stem bark extracts of Berlina grandiflora and one of its active principles, Betulinic acid. *Phytomedicine*.

[209] Shai L. J., Bizimenyera E. S., Bagla V., Mcgaw L. J., Eloff J. N. (2009). Curtisia dentata (Cornaceae) leaf extracts and isolated compounds inhibit motility of parasitic and free-living nematodes. *Onderstepoort Journal of Veterinary Research*.

[210] Chama M. A., Dziwornu G. A., Waibel R. (2016). Isolation, characterization, and anthelminthic activities of a novel dichapetalin and other constituents of Dichapetalum filicaule. *Pharmaceutical Biology*.

[211] Ferreira L. E., Benincasa B. I., Fachin A. L. (2016). Thymus vulgaris L. essential oil and its main component thymol: anthelmintic effects against *Haemonchus contortus* from sheep. *Veterinary Parasitology*.

[212] Banerjee T., Singh A., Kumar S. (2019). Ovicidal and larvicidal effects of extracts from leaves of Andrographis paniculata (Burm. f.) Wall.ex Nees against field isolates of human hookworm (Ancylostoma duodenale). *Journal of Ethnopharmacology*.

[213] Samoylenko V., Dunbar D. C., Gafur M. A. (2008). Antiparasitic, nematicidal and antifouling constituents from juniperus berries. *Phytotherapy Research*.

[214] Qi H., Wang W. X., Dai J. L., Zhu L. (2015). In vitro anthelmintic activity of Zanthoxylum simulans essential oil against *Haemonchus contortus*. *Veterinary Parasitology*.

[215] Van Puyvelde L., Liu M., Veryser C. (2018). Active principles of Tetradenia riparia. IV. Anthelmintic activity of 8(14), 15-sandaracopimaradiene-7*α*, 18-diol. *Journal of Ethnopharmacology*.

[216] Dilrukshi Herath H. M. P., Preston S., Hofmann A. (2017). Screening of a small, well-curated natural product-based library identifies two rotenoids with potent nematocidal activity against *Haemonchus contortus*. *Veterinary Parasitology*.

[217] Liu M., Veryser C., Lu J. G. (2018b). Bioassay-guided isolation of active substances from Semen Torreyae identifies two new anthelmintic compounds with novel mechanism of action. *Journal of Ethnopharmacology*.

[218] Ortu E., Sanna G., Scala A., Pulina G., Caboni P., Battacone G. (2017). In vitro anthelmintic activity of active compounds of the fringed rue Ruta chalepensis against dairy Ewe gastrointestinal nematodes. *Journal of Helminthology*.

[219] Forbes W. M., Gallimore W. A., Mansingh A., Reese P. B., Robinson R. D. (2014). Eryngial (trans-2-dodecenal), a bioactive compound from Eryngium foetidum: its identification, chemical isolation, characterization and comparison with ivermectin in vitro. *Parasitology*.

[220] Williams A. R., Ramsay A., Hansen T. V. A. (2015). Anthelmintic activity of trans-cinnamaldehyde and A-andB-type proanthocyanidins derived from cinnamon (Cinnamomum verum). *Scientific Reports*.

[221] Souza M. M. C., Bevilaqua C. M. L., Morais S. M., Costa C. T. C., Silva A. R. A., Braz-Filho R. (2008). Anthelmintic acetogenin from Annona squamosa L. Seeds. *Anais da Academia Brasileira de Ciências*.

[222] García-Hernández C., Rojo-Rubio R., Olmedo-Juárez A. (2019). Galloyl derivatives from Caesalpinia coriaria exhibit in vitro ovicidal activity against cattle gastrointestinal parasitic nematodes. *Experimental Parasitology*.

[223] Castillo-Mitre G. F., Olmedo-Juárez A., Rojo-Rubio R. (2017). Caffeoyl and coumaroyl derivatives from Acacia cochliacantha exhibit ovicidal activity against *Haemonchus contortus*. *Journal of Ethnopharmacology*.

[224] Castañeda-Ramírez G. S., Torres-Acosta J. F. D. J., Sandoval-Castro C. A. (2019). Bio-guided fractionation to identify Senegalia gaumeri leaf extract compounds with anthelmintic activity against *Haemonchus contortus* eggs and larvae. *Veterinary Parasitology*.

[225] Lin R. J., Chen C. Y., Chung L. Y., Yen C. M. (2010). Larvicidal activities of ginger (Zingiber officinale) against Angiostrongylus cantonensis. *Acta Tropica*.

[226] Jasso Díaz G., Hernández G. T., Zamilpa A. (2017). In vitro assessment of Argemone mexicana, *Taraxacum officinale*, Ruta chalepensis and Tagetes filifolia against *Haemonchus contortus* nematode eggs and infective (L3) larvae. *Microbial Pathogenesis*.

[227] Cavalcante G. S., de Morais S. M., Andre W. P. P. (2016). Chemical composition and in vitro activity of Calotropis procera (Ait.) latex on *Haemonchus contortus*. *Veterinary Parasitology*.

[228] Deepak M., Dipankar G., Prashanth D., Asha M. K., Amit A., Venkataraman B. V. (2002). Tribulosin and *β*-sitosterol-D-glucoside, the anthelmintic principles of Tribulus terrestris. *Phytomedicine*.

[229] Wang G. X., Han J., Zhao L. W., Jiang D. X., Liu Y. T., Liu X. L. (2010). Anthelmintic activity of steroidal saponins from Paris polyphylla. *Phytomedicine*.

[230] Doligalska M., Jóźwicka K., Donskow-Łysoniewska K., Kalinowska M. (2017). The antiparasitic activity of avenacosides against intestinal nematodes. *Veterinary Parasitology*.

[231] Wang X. B., Li G. H., Zheng L. J. (2009). Nematicidal cardenolides from nerium indicum mill. *Chemistry and Biodiversity*.

[232] Satou T., Akao N., Matsuhashi R., Koike K., Fujita K., Nikaido T. (2002). Inhibitory effect of isoquinoline alkaloids on movement of second-stage larvae of Toxocara canis. *Biological and Pharmaceutical Bulletin*.

[233] Paul A., Adnan M., Majumder M. (2018). Anthelmintic activity of Piper sylvaticum Roxb. (family: piperaceae): in vitro and in silico studies. *Clinical Phytoscience*.

[234] Wang G. X., Zhou Z., Jiang D. X. (2010). In vivo anthelmintic activity of five alkaloids from Macleaya microcarpa (Maxim) Fedde against Dactylogyrus intermedius in *Carassius auratus*. *Veterinary Parasitology*.

[235] Ayers S., Zink D. L., Mohn K. (2007). Anthelmintic activity of aporphine alkaloids from Cissampelos capensis. *Planta Medica*.

[236] Barrau E., Fabre N., Fouraste I., Hoste H. (2005). Effect of bioactive compounds from Sainfoin (Onobrychis viciifolia Scop.) on the in vitro larval migration of *Haemonchus contortus*: role of tannins and flavonol glycosides. *Parasitology*.

[237] Wangchuk P., Pearson M. S., Giacomin P. R. (2016). Compounds derived from the Bhutanese daisy, ajania nubigena, demonstrate dual anthelmintic activity against schistosoma mansoni and Trichuris muris. *PLoS Neglected Tropical Diseases*.

[238] Soldera-Silva A., Seyfried M., Campestrini L. H. (2018). Assessment of anthelmintic activity and bio-guided chemical analysis of Persea americana seed extracts. *Veterinary Parasitology*.

[239] Mukai D., Matsuda N., Yoshioka Y., Sato M., Yamasaki T. (2008). Potential anthelmintics: polyphenols from the tea plant Camellia sinensis L. are lethally toxic to *Caenorhabditis elegans*. *Journal of Natural Medicines*.

[240] Kermanshai R., Mccarry B. E., Rosenfeld J., Summers P. S., Weretilnyk E. A., Sorger G. J. (2001). Benzyl isothiocyanate is the chief or sole anthelmintic in papaya seed extracts. *Phytochemistry*.

[241] Navarro-Moll M. C., Romero M. C., Montilla M. P., Valero A. (2011). In vitro and in vivo activity of three sesquiterpenes against L3 larvae of Anisakis type I. *Experimental Parasitology*.

[242] von Son-de Fernex E., Alonso-Díaz M. Á., Valles-de la Mora B., Mendoza-de Gives P., González-Cortazar M., Zamilpa A. (2017). Anthelmintic effect of 2H-chromen-2-one isolated from Gliricidia sepium against Cooperia punctata. *Experimental Parasitology*.

[243] Nguyen B. C. Q., Chompoo J., Tawata S. (2015). Insecticidal and nematicidal activities of novel mimosine derivatives. *Molecules*.

[244] Engström M. T., Karonen M., Ahern J. R. (2016). Chemical structures of plant hydrolyzable tannins reveal their in vitro activity against egg hatching and motility of *Haemonchus contortus* nematodes. *Journal of Agricultural and Food Chemistry*.

[245] Hoste H., Torres-acosta J. F. J. (2011). Veterinary Parasitology Non chemical control of helminths in ruminants adapting solutions for changing worms in a changing world. *Veterinary Parasitology*.

[246] Mukherjee N., Mukherjee S., Saini P., Roy P., P Sinha Babu S. (2016). Phenolics and terpenoids; the promising new search for anthelmintics: a critical review. *Mini-Reviews in Medicinal Chemistry*.

[247] Hirazawa N., Oshima S. I., Mitsuboshi T., Hata K. (2001). The anthelmintic effect of medium-chain fatty acids against the monogenean Heterobothrium okamotoi in the tiger puffer *Takifugu rubripes*: evaluation of doses of caprylic acid at different water temperatures. *Aquaculture*.

[248] Li G.-H., Zhang K. (2014). Nematode-toxic fungi and their nematicidal metabolites. *Nematode-Trapping Fungi*.

[249] Pineda-Alegría J. A., Sánchez J. E., González-Cortazar M. (2020). In vitro nematocidal activity of commercial fatty acids and *β*-sitosterol against *Haemonchus contortus*. *Journal of Helminthology*.

[250] Bizimana N. (1994). Traditional veterinary practice in Africa. *Deutsche Gesellschaft Fur Technische Zusammenarbeit (GTZ)*.

[251] Gachathi F. N. (1989). *Kikuyu Botanical Dictionary of Plant Names and Uses*.

[252] Koko W. S., Galal M., Khalid H. S. (2000). Fasciolicidal efficacy of Albizia anthelmintica and Balanites aegyptiaca compared with albendazole. *Journal of Ethnopharmacology*.

[253] Science and Technology Policy Research Institute, Busia K. (2007). *Ghana herbal pharmacopoeia*.

[254] West African Health Organisation (2013). *West African Herbal Pharmacopoeia*.

[255] Twumasi E. B., Akazue P. I., Kyeremeh K. (2020). Antischistosomal, antionchocercal and antitrypanosomal potentials of some Ghanaian traditional medicines and their constituents. *PLoS Neglected Tropical Diseases*.

[256] Hashmat I., Azad H., Ahmed A. (2012). Neem (azadirachta indica A . Juss) - a nature’s drugstore: an overview. *International Research Journal of Biological Sciences*.

[257] Fomum F. U., Grubben G. J. H., Denton O. A. (2004). PROTA4U vernonia amygdalina delile. *Ressources Végétales de l’Afrique Tropicale*.

[258] Chhabra S. C., Mahunnah R., Mshiu E. (1991). Plants used in traditional medicine in eastern Tanzania. V. Angiosperms (passifloraceae to sapindaceae). *Journal of Ethnopharmacology*.

